# Diagnostic imaging and emerging image-guided neurointervention in brain tumors: a narrative review

**DOI:** 10.3389/fradi.2026.1914188

**Published:** 2026-09-08

**Authors:** Zahin Alam, Triston Messer, Anirudh Maddali, Jake Heath, Kush Desai, Srihari Sundararajan, David Monoky, Brij Reddy

**Affiliations:** 1 Department of Radiology, Hackensack Meridian School of Medicine, Nutley, NJ, United States; 2 Department of Interventional Radiology, Robert Wood Johnson Medical School, New Brunswick, NJ, United States; 3 Department of Radiology, Hackensack University Medical Center, Hackensack, NJ, United States; 4 Clearview Radiology PC, Bayside, NY, United States

**Keywords:** brain tumors, diagnostic neuro-imaging, image-guided intervention, image-guided neuro-oncology, interventional neuroradiology, neuro-oncology, neuroradiology, neurosurgery

## Abstract

Brain tumors are heterogeneous neoplasms requiring multidisciplinary management and precise diagnostic and therapeutic strategies. Diagnostic radiology plays a central role in tumor detection, characterization, treatment planning, and surveillance through advanced imaging modalities such as multiparametric magnetic resonance imaging, perfusion imaging, diffusion techniques, spectroscopy, and molecular imaging. Concurrently, image-guided procedures performed across endovascular neurointerventional and neurosurgical workflows, including embolization, laser interstitial thermal therapy, intra-arterial chemotherapy, convection-enhanced delivery, and blood-brain barrier modulation, are expanding minimally invasive options in selected patients. This review synthesizes current and emerging diagnostic radiology and image-guided approaches across major brain tumor types, including glioblastoma, low-grade glioma, metastases, primary central nervous system lymphoma, and meningioma. Furthermore, this review highlights the growing overlap among diagnostic radiology, interventional radiology, and neurosurgical workflows, emphasizes the increasingly central role of imaging in guiding therapy, and identifies the need in which coordinated multidisciplinary workflows should be prospectively evaluated as diagnostic and therapeutic technologies continue to converge. Lastly, this review distinguishes established clinical tools from selectively used and investigational approaches by summarizing evidence level, patient-selection considerations, and barriers to routine implementation.

## Introduction

Brain tumors encompass a diverse group of neoplasms with significant clinical and societal impact, accounting for significant morbidity and mortality. Epidemiological analyses show increased incidence and prevalence of these neoplasms, particularly in high sociodemographic regions, with glioblastoma and meningioma being the most common malignant and non-malignant subtypes, respectively (1, 2). Overall disease burden is amplified by the complex diagnoses, variable disease presentations, and requirement of multidisciplinary management.

Diagnostic radiology (DR) is a core component of treating patients with brain tumors, providing vital information for initial detection, differential diagnosis, tumor characterization, treatment planning, and longitudinal follow-up. The American College of Radiology emphasizes use of advanced modalities such as MRI, perfusion imaging, MR spectroscopy, and PET for anatomical and functional assessment, surgical planning, and follow-up surveillance (3). This allows for accurate demarcation of tumor margins, detailed analyses of molecular and genetic characteristics, and evaluation of therapeutic response.

Image-guided neuro-oncology encompasses several related but distinct domains. Diagnostic neuroradiology provides anatomic, physiologic, metabolic, and molecular imaging for diagnosis, treatment planning, and surveillance. Endovascular neurointervention includes diagnostic angiography, preoperative tumor embolization, and selected intra-arterial therapeutic-delivery or blood-brain barrier (BBB) modulation procedures. Neurosurgery-led image guided procedures include stereotactic biopsy, intraoperative MRI-guided resection, laser interstitial thermal therapy (LITT), and most convection-enhanced delivery (CED) procedures (4–7).

Combining diagnostic neuroradiology, endovascular neurointervention, and neurosurgery-led image-guided perspectives is justified by rapid technological advancement and concomitant intersection of advanced imaging and interventional techniques in neuro-oncology (6). Despite this evolution, there remains limited literature synthesizing multidisciplinary workflows into a cohesive framework. Importantly, many contemporary image-guided interventions in neuro-oncology, including laser interstitial thermal therapy and catheter-based regional therapies, are performed by neurosurgeons but rely fundamentally on advanced imaging platforms. As such, these interventional neuro-oncology procedures further underscore the need for an integrated imaging–intervention paradigm. Therefore, a unified synthesis of current and emerging neuroradiologic and image-guided protocols may enhance diagnostic accuracy, improve treatment planning, and support multidisciplinary decision-making, particularly in complex cases with evolving tumor pathologies (8).

This review will examine the use of diagnostic neuroradiology, endovascular neurointervention, and neurosurgery-led image-guided procedures across major common brain tumor types, including glioblastoma, low-grade glioma, brain metastases, central nervous system lymphoma, and meningioma, highlighting the diversity of disease processes encountered in current neuro-oncologic practice (1–5). This review is intended to provide a broad, clinically oriented overview for a general neurology and multidisciplinary neuro-oncology audience rather than a subspecialty-level technical discussion for neuroradiologists or neuro-oncologists. Accordingly, we do not propose a prescriptive or universally applicable operational workflow. Instead, across major brain tumor types, we map how diagnostic neuroradiology, endovascular neurointervention, and neurosurgery-led image-guided procedures currently intersect and identify a need in which more coordinated workflows should be prospectively evaluated. Throughout the review, “image-guided neuro-oncology” is used as the multidisciplinary umbrella term, whereas the primary procedural specialty is stated for individual techniques.

### Methodology and literature selection

This narrative review was developed with reference to the Scale for the Assessment of Narrative Review Articles (SANRA) (9). The literature search was initiated in December 2025 and updated iteratively through July 31, 2026. PubMed/MEDLINE, Scopus, and Google Scholar were searched from database inception through July 31, 2026. Search terms included glioblastoma, low-grade glioma, brain metastases, primary central nervous system lymphoma, and meningioma as well as terms related to diagnostic imaging and image-guided intervention, including MRI, diffusion, perfusion, spectroscopy, PET, radiomics, artificial intelligence, intraoperative MRI, LITT, CED, focused ultrasound, blood–brain barrier disruption, intra-arterial therapy, stereotactic biopsy, and embolization. Complete database-specific search strategies are provided in Supplementary Table S1.

English-language peer-reviewed clinical studies, reviews, consensus statements, and professional-society guidelines relevant to diagnosis, treatment planning, image-guided intervention, or surveillance were considered. Recent guidelines and clinical studies were prioritized, while older foundational studies were retained when relevant. Studies without a clear neuro-oncologic application were excluded, and reference lists of selected articles were reviewed for additional sources. Conference abstracts and trial registrations were included only when describing preliminary or ongoing approaches and were identified accordingly. As this was a narrative review, formal dual-reviewer screening and standardized risk-of-bias assessment were not performed.

## Discussion

### Glioblastoma

#### Conventional imaging

**Figure 1:**
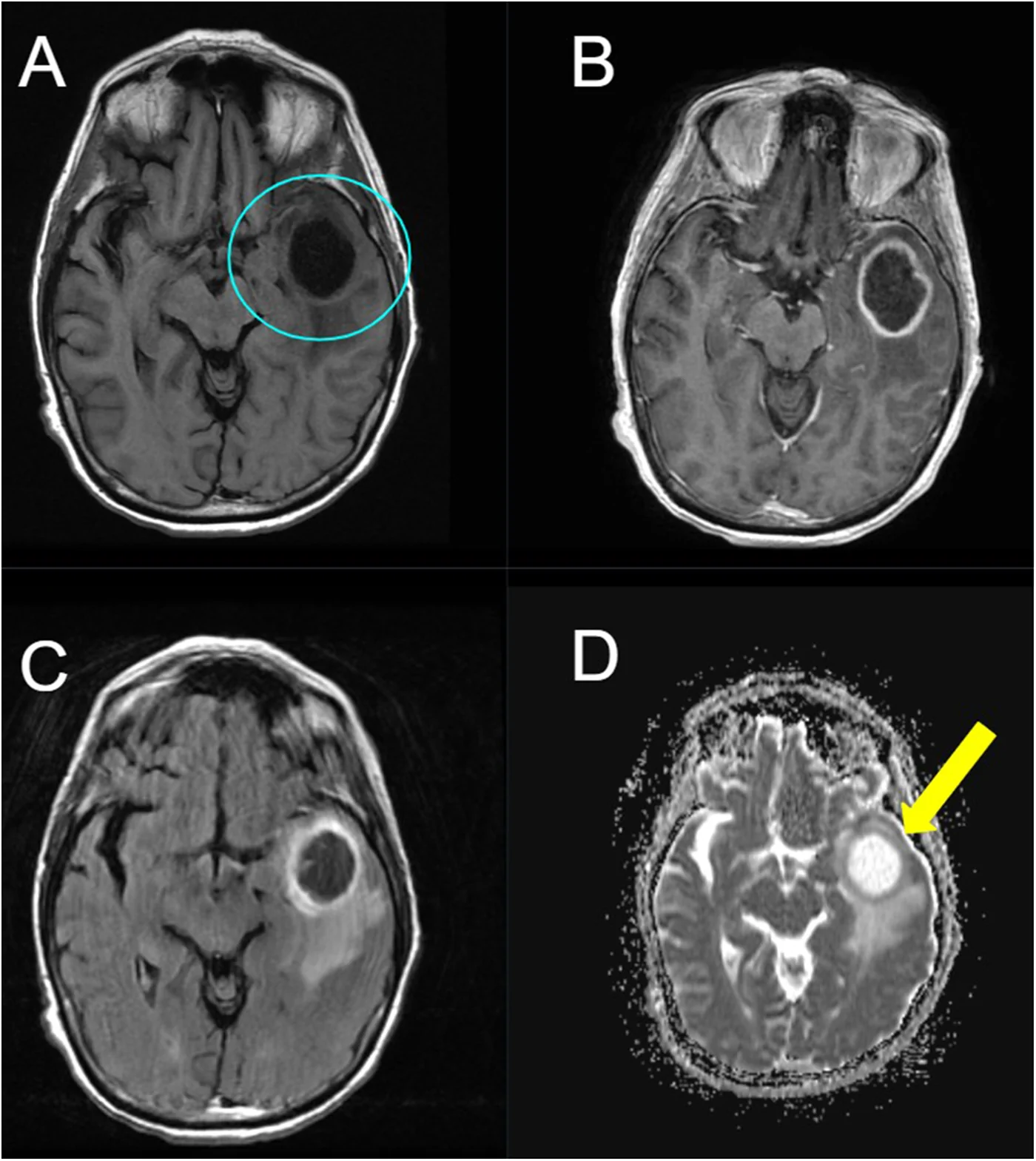
Glioblastoma. **(A)** Axial T1-weighted pre-contrast MRI shows a centrally T1 hypointense intraaxial mass centered within the left temporal lobe (Blue circle). **(B)** Axial T1-weighted post-contrast image demonstrates central hypointensity consistent with necrotic/cystic change, surrounded by thick, irregular peripheral ring enhancement. **(C)** Axial FLAIR image demonstrates associated surrounding FLAIR hyperintense edema. **(D)** ADC map shows low ADC values along the peripheral rim suggesting high cellularity and a higher-grade lesion. The central necrotic component demonstrates increased ADC values (yellow arrow).

**Figure 2:**
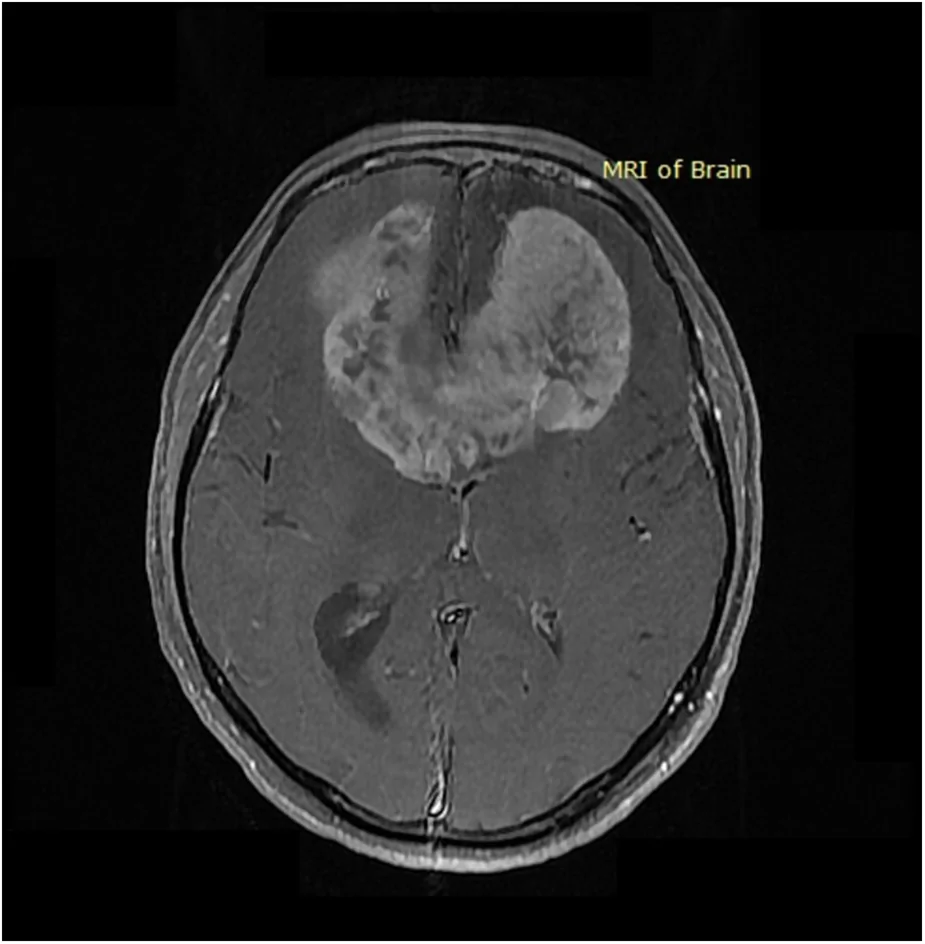
Butterfly glioblastoma. Axial T1-weighted post-contrast MRI demonstrates a heterogeneously enhancing “butterfly” lesion centered in the frontal lobes, crossing the genu of the corpus callosum. The lesion exhibits diffuse heterogeneous enhancement and central necrosis, imaging features characteristic of a high-grade glioma.

Glioblastoma refers specifically to an adult, IDH-wildtype, WHO grade 4 neoplasm. IDH-wildtype glioblastomas represent approximately 90% of adult glioblastomas and correspond to the entity historically regarded as “primary” glioblastoma. These tumors exhibit a more aggressive biological behavior and are associated with a poorer prognosis. In contrast, tumors previously referred to as “secondary glioblastomas” are now classified as Astrocytoma, IDH-mutant, CNS WHO grade 4. Although both entities are grade 4 diffuse gliomas, IDH-mutant astrocytomas typically occur in younger adults and are associated with a more favorable prognosis. Under this current framework, a definitive diagnosis can be established through two distinct pathways. Traditionally, tumor diagnosis is achieved with identification of classic histopathological features, namely necrosis and/or microvascular proliferation, within an IDH-wildtype diffuse astrocytic tumor. Alternatively, in the absence of these high-grade histological features, an adult-type IDH-wildtype diffuse astrocytic glioma can be upgraded to a CNS WHO grade 4 glioblastoma if it possesses any one of three specific molecular markers: TERT promoter mutation, EGFR gene amplification, or combined chromosome 7 gain and chromosome 10 loss (+7/−10). It is critical to distinguish these autonomous molecular upgrading criteria from other genetic alterations like CDKN2A/B homozygous deletion; while CDKN2A/B deletion serves to upgrade IDH-mutant astrocytomas to grade 4, it is not a standalone diagnostic marker for IDH-wildtype glioblastoma (10). In respect to conventional imaging, due to the large size of glioblastomas at time of diagnosis, MRI estimates tumor size by visualizing gadolinium contrast that extravasates through tumor vasculature (11). MRI features demonstrate a heterogeneously enhancing mass on post-contrast T1-weighted imaging, with central necrosis or hemorrhagic components in tumor core and surrounding vasogenic edema visible on T2/FLAIR hyperintensity (12, 13). On MRI, glioblastomas often exhibit irregular margins and significant mass effect, with infiltration into adjacent brain parenchyma (Figures 1, 2) (14).

#### Advanced and molecular imaging

On perfusion MRI, especially dynamic susceptibility contrast (DSC), relative cerebral blood volume (rCBV) is elevated in high-grade gliomas such as glioblastoma, often secondary to increased levels of neovascularization. rCBV is valuable in distinguishing glioblastoma from other masses such as primary CNS lymphoma, which typically shows low rCBV (3, 15). Perfusion metrics provide prognostic utility, correlating with survival rates and guiding biopsy targeting (16). Moreover, Johnson et al., in a systematic review, highlights that perfusion MRI can be utilized to differentiate true tumor progression from treatment-related imaging changes or pseudo-progression in suspected progressive glioblastoma (17).

Magnetic resonance spectroscopy (MRS) provides chemical and metabolic information regarding brain masses such as glioblastoma through comparison of N-acetylaspartate (NAA) with choline. Typically, the amounts of NAA are greater than choline in normal brain tissue, however glioblastoma commonly shows increased choline and decreased N-acetylaspartate, reflecting increased membrane turnover and neuronal loss respectively (18). Diffusion weighted imaging (DWI) along with apparent diffusion coefficient (ADC) mapping can analyze cellular density for solid tumors like glioblastoma. Specifically, glioblastoma often demonstrates low ADC values in solid tumor regions, which correlates with high cellular density. DWI with ADC mapping is useful for tumor grading and to distinguish glioblastoma from metastases and lymphomas (15). DWI is mainly used as the imaging method to detect progression of previously diagnosed glioblastoma (17).

Treatment response in adult diffuse gliomas is commonly evaluated using criteria developed by the international Response Assessment in Neuro-Oncology (RANO) working group. RANO 2.0 is the principal MRI- and clinically based framework for assessing treatment response in adult high- and lower-grade diffuse gliomas. It evaluates enhancing and nonenhancing tumor components while incorporating clinical status, corticosteroid use, baseline imaging, and confirmatory imaging when apparent progression may represent pseudoprogression (19). In contrast, PET RANO 1.0 is the first dedicated framework for amino acid PET-based response assessment and provides complementary criteria for evaluating metabolic response, stable disease, and progression (20). Amino acid PET tracers, including ^18F-fluoroethyl-L-tyrosine (^18F-FET), ^18F-fluoro-L-dihydroxyphenylalanine (^18F-FDOPA), and ^11C-methionine (^11C-MET), may complement MRI for tumor delineation, biopsy or treatment targeting, and differentiation of recurrent glioma from treatment-related changes (21). However, tracer availability, acquisition protocols, and interpretive thresholds vary across institutions.

In review of emerging diagnostic therapies, radiomics and AI have shown promising results for predicting treatment response and survival rates in glioblastoma, specifically when applied to advanced MRI techniques. Radiomics extracts quantitative features from routine and advanced MRI to capture features like tumor shape, intensity, texture, and heterogeneity beyond visual assessment. Multiparametric models have shown potential in tracking changes in glioblastoma features over time, thus predicting survival rates, progression, and treatment susceptibility (22). Dedhia et al., in a review consisting of 52 studies and 12,482 patients, found that radiomics improve initial diagnostic assessment, distinguish true progression, and enable radiogenomic inference of key molecular signatures without biopsy (23). Though not yet incorporated wide-scale clinically, progress in radiomics and artificial intelligence can integrate multiple imaging modalities to yield higher diagnostic accuracy and more optimal treatment monitoring for brain tumors and other pathologies. However, most reported models remain retrospective, and routine adoption is limited by heterogeneous acquisition protocols, segmentation variability, limited external validation, and uncertain effects on clinical decision-making.

#### Image-guided procedures

Image-guided techniques are foundational to contemporary glioblastoma management. While primary treatment of glioblastoma consists of neurosurgical resection, selected image-guided procedures may be considered for recurrent, deep-seated, or surgically inaccessible disease. Endovascular specialists may perform selected preoperative embolization or intra-arterial procedures, whereas stereotactic biopsy, intraoperative MRI-guided resection, LITT, and most CED procedures are primarily neurosurgery-led. Although preoperative embolization is not routinely indicated for glioblastoma due to its typically modest vascularity, a small recent series suggests that selective embolization of dominant feeders may be beneficial in carefully chosen cases. In a 2026 series, Uchida et al. demonstrated that preoperative embolization of glioblastoma feeding arteries provided a clearer surgical field, reduced intraoperative bleeding, and facilitated more precise tumor localization without neurological complications, supporting a possible adjunctive role that requires confirmation in larger comparative cohorts (24).

Intraoperative MRI (iMRI) is an established neurosurgical adjunct used at selected centers during glioma resection. During surgery, opening the dura, draining cerebrospinal fluid, changes in edema, and removing tumor tissue can cause the brain to shift from its position on preoperative imaging. As a result, neuronavigation based only on preoperative scans may become progressively less accurate as the operation continues. Intraoperative MRI provides updated anatomic information that can identify residual tumor, correct neuronavigation, and guide additional resection. Although iMRI may improve the extent of resection, its effects on long-term survival and costeffectiveness remain less certain. Although iMRI may improve the extent of resection, its effects on long-term survival and cost-effectiveness remain less certain. Important limitations include capital and maintenance costs, limited availability of MRI-equipped operating rooms, the need for MRI-compatible instruments and trained personnel, anesthesia and workflow complexity, additional operative time, and image artifacts (25).

In contrast to iMRI-guided resection, which supplements open surgery, MR-guided LITT is a minimally invasive neurosurgery-led option used in selected patients with recurrent, deep-seated, or surgically inaccessible tumors when open resection is not feasible or carries substantial morbidity. LITT involves the insertion of a probe, thermometric calibration, and subsequent energy modulation under MR guidance (26). Overall, LITT grants the ability for physicians to utilize predictive modeling that permits voxel-level temperature mapping, thus improving ablation uniformity and safety, especially in intricate area lesions. Studies utilizing LITT have achieved results with as high as an average of 93% of tumor volume treatment in glioblastoma (27). Available evidence is derived largely from retrospective cohorts, single-center series, and heterogeneous systematic reviews as randomized comparisons and standardized selection criteria remain limited (26, 28–30). Interestingly, analysis has shown that in patients with surgically accessible, unifocal first-time recurrent glioblastoma, LITT demonstrated comparable overall and progression-free survival to repeat surgical resection while offering significantly shorter hospital stays (31).

Convection enhanced delivery (CED) is an emerging investigational technique that enables direct parenchymal infusion of therapeutic agents, bypassing the blood-brain barrier. Utilizing MR- or CT-guided catheter systems, clinicians can microinfuse treatments such as liposomal or nanoparticle agents, immunotoxins and viral vectors directly into the tumor (32). These intratumoral distributions are verified in real time through the utilization of MR perfusion and diffusion sequences, with the added benefits of a computational modeling system that actively predicts and minimizes backflow or leakage along the catheter tract (33). Early results and reviews have investigated the use of CED for glioblastoma, with promising findings (34–36). In 2022, Spinazzi et al. reported the first in-human experience of CED of chemotherapy for recurrent glioblastoma. Their findings demonstrated feasibility of CED, with evidence of sustained intratumoral drug distribution and acceptable safety (36, 37). However, CED possesses limitations, including the heterogeneity of flow through tissues and variability in catheter dwell time, which has led to further research and development into improved catheter designs, such as reflux-resistant tips and optimized infusion systems, to optimize delivery and reduce complications (38).

Other experimental modalities include focused ultrasound mediated blood barrier opening, which aim to generate a transient opening for therapeutic treatments to enter the brain (39, 40). Microbubble-enhanced transcranial focused ultrasound (MB-FUS) facilitates controlled and localized blood-brain barrier disruption for enhanced drug delivery (41, 42). First, a 2021 Phase 0 (NCT03322813) MB-FUS clinical trial demonstrated its potential to safely open the BBB and improve drug delivery to gliomas (43). Subsequently, a 2025 multicenter Phase 1/2 study reported favorable safety and longer observed overall survival when MB-FUS was combined with temozolomide than in matched controls (39). Animal analyses support these phase 1/2 trial results (44). Treatment remains investigational, and unresolved barriers include skull-dependent acoustic transmission, access to specialized devices, repeated-treatment logistics, and the need for phase III comparative evidence.

Endovascular neurointerventional approaches are being investigated as regional adjuncts to systemic therapy for glioblastoma. Intra-arterial drug delivery represents an investigational strategy for delivery of chemotherapy directly through cerebral arteries, thus achieving higher tumor drug concentrations with limited systemic toxicity (45). Most recently, a 2026 single-center series of 70 patients with newly diagnosed or recurrent glioblastoma found that superselective intra-arterial cerebral infusion of bevacizumab or cetuximab following osmotic blood–brain barrier disruption was feasible and safe, with high procedural success, short hospitalizations, low complication rates, and no procedure-related deaths (46). A 2025 computer-modeling study evaluated superselective intra-arterial yttrium-90 delivery for recurrent glioblastoma (47). Two registered early-phase trials are evaluating intra-arterial cetuximab in newly diagnosed glioblastoma and temsirolimus in recurrent high-grade glioma (48, 49). These trial registrations describe ongoing investigations and are not presented as evidence of clinical efficacy. Along with treatment use, intra-arterial chemoperfusion was shown to be a safe palliative treatment in patients with primary brain malignancies (predominantly glioblastoma) with no major adverse events (50). Despite the growing body of studies, systemic reviews have shown that while intra-arterial delivery is safe and has the potential to achieve higher local drug concentrations for glioblastoma, the scarcity of phase III trials limits the ability to definitively conclude therapeutic benefit (45).

#### Evidence, limitations, and patient selection

Conventional MRI and diffusion imaging have established clinical roles in glioblastoma management, whereas perfusion imaging, MR spectroscopy, and amino acid PET are used selectively depending on the diagnostic question and institutional expertise. iMRI and LITT are selectively used at specialized centers, particularly when lesion location, prior therapy, or operative risk limits conventional resection. CED, focused-ultrasound blood-brain barrier opening, glioblastoma embolization, and intra-arterial drug delivery remain investigational because evidence is dominated by early-phase trials, retrospective series, or nonrandomized comparisons. Patient selection should account for lesion size and location, mass effect, vascular anatomy, prior radiation, steroid dependence, performance status, and the availability of multidisciplinary expertise.

#### Future directions

Future studies should prospectively compare emerging imaging and image-guided approaches with standard of care. Studies should use consistent MRI protocols and clear definitions of tumor progression, pseudoprogression, treatment response, and local control to properly compare across institutions. Investigators should report how image findings affected treatment type selection or procedural planning, such as biopsy targeting, catheter placement, ablation margins, or extent of resection. Most importantly, research should highlight whether these changes improve tumor control, neurologic function, survival, or quality of life.

### Low-grade gliomas (LGGs)

#### Conventional imaging

**Figure 3:**
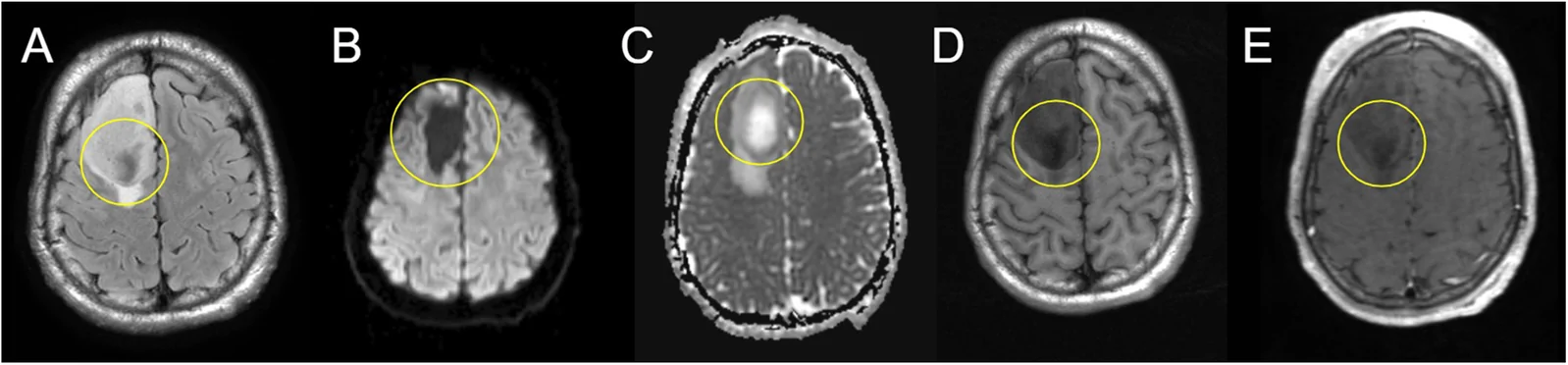
Low-grade glioma. **(A)** Axial FLAIR demonstrates a hyperintense intraaxial lesion within the high right anterior frontal lobe (yellow circle). **(B)** Axial DWI reveals no restriction (yellow circle). **(C)** ADC map shows higher value of the lesion (yellow circle). **(D)** Axial T1-weighted pre-contrast MRI shows hypointense lesion (yellow circle). **(E)** Axial T1-weighted postcontrast MRI shows no abnormal enhancement (yellow circle).

Although “low-grade glioma” remains a commonly used umbrella term in clinical practice, it encompasses a heterogeneous group of tumors with distinct histologic and molecular classifications. Under the 2021 WHO classification, adult-type diffuse gliomas are divided into astrocytoma, IDH-mutant, CNS WHO grades 2–4; oligodendroglioma, IDH-mutant and 1p/19q-codeleted, CNS WHO grades 2–3; and glioblastoma, IDH-wildtype, CNS WHO grade 4. Therefore, “low-grade glioma” remains a clinically useful but imprecise term and should not replace an integrated histologic and molecular diagnosis (51). For the purposes of this review, we use the term broadly to refer to infiltrative gliomas with relatively indolent imaging features, while acknowledging the limitations of this terminology in modern CNS tumor classification (52). These tumors often affect pediatric and young adult populations, and are characterized by an indolent clinical course, manifesting symptomatically with seizures, headaches, or focal neurologic deficits (52). On MRI, low-grade gliomas typically appear as T2/FLAIR hyperintense lesions with little to no contrast enhancement, minimal mass effect, and no significant necrosis or hemorrhage (Figure 3). LGGs generally lack the aggressive imaging features more characteristic of higher-grade gliomas, such as marked enhancement, central necrosis, hemorrhage, or substantial vasogenic edema (53). These imaging characteristics reflect the relatively preserved blood-brain barrier and slower growth of many low-grade lesions.

#### Advanced and molecular imaging

The main challenge in managing LGGs is delayed anaplastic progression. Thus, advanced imaging techniques play a critical role in early identification of infiltrative involvement, improving diagnosis, characterization, and treatment planning (54–56). Functional MRI can localize cortex adjacent to the lesion, while diffusion tensor imaging and tractography assess involvement or displacement of important white matter tracts (53, 55, 57). MR spectroscopy generates essential metabolic information, often demonstrating elevated choline with relatively preserved N-acetylaspartate, displaying a low-grade tumor profile and helping guide biopsy targeting and longitudinal surveillance (53, 55, 57, 58). An important role of radiologists in the management of LGGs is monitoring for anaplastic progression by identifying interval changes on serial imaging, including the development of new or increasing contrast enhancement, accelerated tumor growth, rising perfusion metrics, restricted diffusion, and evolving metabolic abnormalities on MR spectroscopy.

Radiogenomics, an emerging modality, identifies correlations between imaging phenotypes and genetic data, and has been shown to enhance LGG diagnostics. Radiogenomics can evaluate low-grade gliomas by correlating MRI phenotypic characteristics with key molecular alterations in LGGs such as IDH mutation status, 1p/19q codeletion, and MGMT promoter methylation (59). However, these models may not perform reliably across institutions because most were developed retrospectively, used nonstandardized image-processing methods, and have undergone limited prospective external validation.

As IDH status is central to the classification and management of adult-type diffuse gliomas, noninvasive molecular characterization has become an important imaging research focus. Conventional MRI phenotypes, diffusion and perfusion metrics, MR spectroscopy, and radiomic models can provide complementary information regarding IDH status, 1p/19q codeletion, and tumor grade. Quantitative proton MR spectroscopy may directly detect the oncometabolite 2-hydroxyglutarate produced by mutant IDH enzymes, supporting preoperative stratification and longitudinal metabolic assessment in selected centers (60). Spherical mean MRI is a newer diffusion-based approach designed to reduce the influence of microscopic orientation dispersion and characterize tumor microstructure. A recent prospective study reported promising performance for predicting IDH status and tumor grade compared with conventional ADC measurements (61). These results require multicenter validation across scanners, acquisition protocols, molecular subgroups, and treatment states before routine implementation. At present, MRI based molecular prediction should be viewed as a complementary tool for preoperative counseling, biopsy planning, and research stratification rather than a replacement for integrated histopathologic and molecular diagnosis. Important barriers include small or imbalanced datasets, model overfitting, scanner and protocol variability, and limited external and prospective validation.

#### Image-guided procedures

With respect to low grade gliomas, image-guided intervention currently focuses on individualized, minimally invasive management utilizing diagnostic imaging as the platform for the targeted therapy. MR-guided LITT may provide a minimally invasive cytoreductive option for careful selected deep-seated or surgically challenging lesions when open resection carries substantial risk (62). During LITT for LGG, the multidisciplinary team (consisting of neurosurgeons, neuro-oncologists, and neuroradiologists) manages the laser catheter trajectory using real-time feedback. Small institutional cohorts have reported radiographic stability, seizure improvement, and short hospital stays, but the magnitude and durability of benefit remain uncertain (63). This integration of real-time imaging is critical to treatment success, as the perfusion maps verify viable tumor tissue and the diffusion tensor imaging helps ensure physicians are able to avoid major tracts during trajectory planning. The National Comprehensive Cancer Network (NCCN) guidelines now include LITT as a category 2B recommendation for patients who are poor surgical candidates for craniotomy or resection (64). Real-time MRI thermometry is critical for enabling precise ablation control and monitoring thermal damage during LITT procedures (65). Several small scale studies have found promising results of LITT-based therapy on LGG management, especially in pediatric populations (66–68). As pediatric low-grade gliomas are biologically and molecularly distinct from adult-type diffuse gliomas, pediatric LITT studies are included here as supportive evidence of technical feasibility and early outcomes but should not be directly generalized to adult populations (69). In the adult population, a 2026 pilot study comparing LITT to surgical resection for newly diagnosed deep-seated LGG reported similar observed progression-free and overall survival, but the small nonrandomized design limits conclusions (70). Thus, LITT should be considered a selective option rather than a replacement for maximal safe resection in otherwise appropriate surgical candidates, though LITT does show promise. As introduced in the section prior, emerging research has shown potential for radiomics, radiogenomics, and machine learning for predicting molecular subtypes, tumor grade, and prognosis of LGG (71). Of particular note, a deep learning–assisted radiomics model integrating clinical and preoperative MRI data from 349 LGG patients reported predictive performance of overall survival, time to malignant transformation, and tumor growth velocity in both preoperative and postoperative settings (72). Radiomics-driven infiltration modeling may eventually assist trajectory or treatment-zone planning, but this application remains investigational and has not yet demonstrated improved clinical outcomes. Additional studies are needed to explore this avenue. Additionally, neurointerventional oncology is exploring AI-based heat-distribution prediction modeling during LITT procedures (73, 74). One important objective of advanced thermal modeling is to address the “heat sink effect,” where highly vascular regions can dissipate heat and reduce effectiveness of LITT (75). Conceptually, with the integration of AI in real-time thermometry, real-time feedback on tumor changes may potentially promote radiation dose adaptation and increased precision. However, generalizability would be limited by retrospective AI model development, heterogeneous image preprocessing, and limited prospective external validation.

#### Evidence, limitations, and patient selection

Serial conventional and advanced MRI are established components of LGG management, while 2-hydroxyglutarate spectroscopy and MRI-based molecular prediction remain specialized or investigational. LITT may be reasonable for selected deep, recurrent, or surgically high-risk lesions, but evidence is based mainly on small retrospective or institutional cohorts. Patient selection should account for tumor location, growth trajectory, seizure burden, molecular diagnosis, expected resection morbidity, thermal safety margins, and overall need for tissue acquisition.

#### Future directions

Future prospective, multicenter studies should first determine how accurately imaging-based molecular predictions reflect tissue-based diagnoses, including IDH status and tumor grade. Beyond diagnostic accuracy, these studies should evaluate whether incorporating molecular imaging into clinical practice improves biopsy targeting, surgical planning, and surveillance strategies. Ultimately, the value of these tools will depend on whether they meaningfully improve treatment selection, disease monitoring, and patient outcomes.

### Metastatic brain disease

#### Conventional imaging

**Figure 4:**
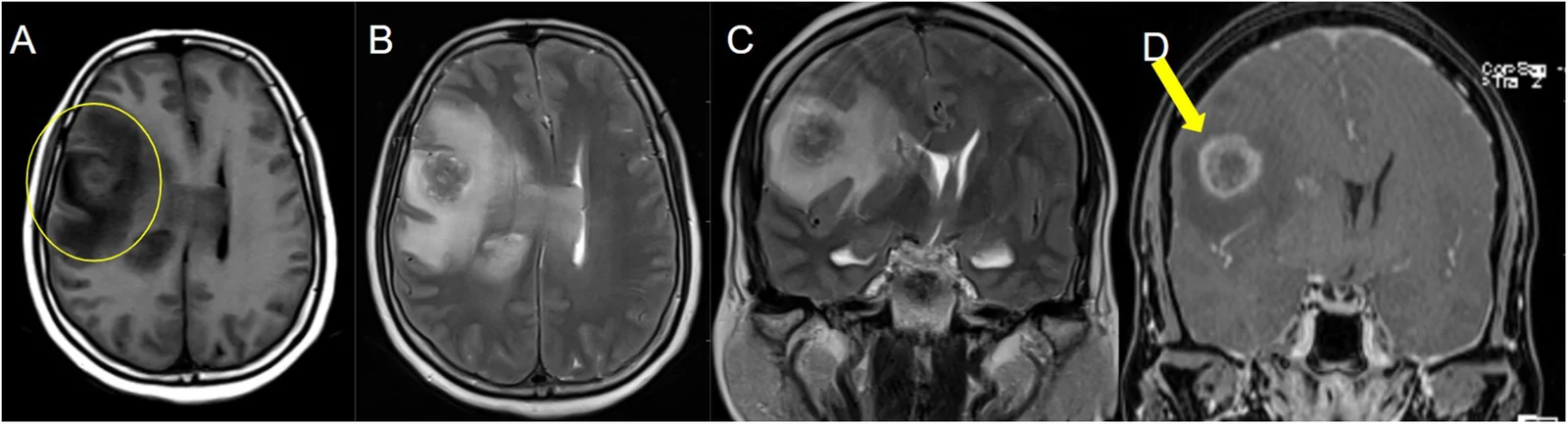
Brain metastases. **(A)** Axial T1-weighted image shows a necrotic, T1 hypointense lesion in the right frontal lobe with extensive surrounding vasogenic edema (yellow circle). There is significant mass effect on adjacent structures with leftward midline shift. A smaller lesion is noted along the posteromedial aspect of the primary mass. **(B)** Axial T2-weighted image demonstrates a predominantly T2 hyperintense lesion with extensive surrounding hyperintense vasogenic edema. **(C)** Coronal T2-weighted image better delineates the degree of mass effect and leftward midline shift. **(D)** Coronal T1-weighted post-contrast image demonstrates irregular thick peripheral ring enhancement of the dominant lesion (yellow arrow), with a smaller enhancing lesion medially suggestive of metastatic disease.

Metastatic brain disease is the most frequent intracranial malignancy in adults, with common sources originating from primary tumors of the lung, breast, melanoma, kidney, and gastrointestinal tract (76). Metastases classically present as multiple, well-circumscribed enhancing lesions, with predilection for gray-white matter junction (Figure 4) (77). However, brain metastases can present as solitary brain lesions, posing diagnostic challenges due to mimicry of other primary brain lesions such as glioblastoma (78–80). Typical lesion characteristics are associated with surrounding vasogenic edema on T2-weighted and FLAIR sequences. Hemorrhagic components may be seen depending on the primary malignancy (81). In certain cases, diagnostic uncertainty may be introduced due to imaging feature overlap with alternative considerations, including enhancing foci of demyelination, microabscesses, and subacute infarction.

#### Advanced imaging and molecular imaging

Advanced imaging techniques, such as Perfusion MRI and diffusion-weighted imaging, are valuable in differentiating tumor recurrence from radiation necrosis, as recurrent metastatic lesions feature elevated relative cerebral blood volume and variable diffusion restriction compared with radiation treatment-related changes (83). MR spectroscopy may further aid differentiation, with recurrent disease typically showing elevated choline and reduced N-acetylaspartate, whereas radiation necrosis often demonstrates lipid-lactate peaks and lower choline levels (84). However, no single imaging parameter can definitively distinguish recurrent metastasis from treatment-related change, thus interpretation should integrate findings across multiple imaging sequences, changes on serial examinations, and the timing of abnormalities relative to treatment.

PET imaging, in a complementary role, provides more utility with systemic staging, identification of primary malignancy, and longitudinal care than for characterization of intracranial lesions themselves (85). The Brain Metastases Response Assessment in Neuro-Oncology (RANO-BM) criteria, developed through an international multidisciplinary effort, provide a standardized framework for evaluating intracranial response and progression using lesion measurements together with clinical status and corticosteroid use (86). Although FDG-PET remains useful for systemic staging and identification of a primary malignancy, amino acid PET with ^18F-FET, ^18F-FDOPA, or ^11C-MET may provide complementary information when MRI remains equivocal for recurrent metastasis versus treatment-related change. Recent joint EANM/EANO/RANO/SNMMI guidance supports amino acid PET as an adjunct to MRI in this setting, although tracer access and interpretive standardization remain variable (87).

Lastly, AI-assisted detection and longitudinal segmentation are promising, but most systems require additional multicenter validation and assessment of false-positive burden (88, 89).

#### Image-guided procedures

In contrast to glioblastomas and low-grade gliomas, which are typically not sufficiently hypervascular to warrant preoperative embolization, endovascular neurointervention contributes to brain metastasis management primarily through selective preoperative embolization of hypervascular tumors and adjunctive ablation techniques that enhance surgical safety. Hypervascular lesions, especially those originating from primary renal cell carcinoma, thyroid cancer, and choriocarcinoma cancers, are targeted (90). Metastases from hypervascular primary tumors can present major operative challenges such as an increased risk of intraoperative hemorrhage and limited surgical visualization (91). The best candidates for endovascular embolization are lesions with angiographically evident tumor blush (92). Procedural planning begins with high-quality anatomic imaging followed by diagnostic angiography to define dominant vascular supply, variant anatomy, anastomoses, and venous drainage relevant to hemorrhage risk (92). Super-selective catheterization under fluoroscopic- or CT-guidance enables precise deposition of particles, coils, or liquid embolics. The objective is devascularization of the hypervascular lesions while minimizing nontarget embolization in collateral pathways with the external and internal carotid arteries. Thus, embolization carries risk for ischemic injury resulting in visual loss or cranial nerve neuropathies, necessitating proper pre-procedural planning (93).

Similar to its role in other brain tumor pathologies, LITT is a selectively used, neurosurgery-led image-guided option in the management of brain metastases, with particular utility for deep-seated or surgically inaccessible lesions (94). Additionally, the NCCN recognizes LITT as a valuable treatment option for recurrent metastases and radiation necrosis, particularly in patients who are poor surgical candidates, given its ability to provide minimally invasive cytoreduction and alleviate mass effect (94). Critical to this approach are quantitative imaging biomarkers, including tracking apparent diffusion coefficient and perfusion weight signals, allowing for efficacious ablation. There are limitations to the current capabilities of LITT however, as there is often incomplete ablation of lesions larger than 3 cm and the need for short-term corticosteroid taper to treat perilesional edema (62). Thus, like traditional embolic treatments for brain metastases, existing data supports LITT as a technique for well-mapped cases but not a universal solution. ASCO-SNO-ASTRO guidelines state that while LITT is an emerging technology developed for local control and management of radiation necrosis, without prospective randomized trial data, no recommendation can be made for or against LITT, and its role in brain metastases treatment continues to be explored (95).

Investigational endovascular and image-guided approaches include blood-brain barrier modulation and targeted intra-arterial therapy, which serve to maximize first-pass delivery to lesions while reinforcing the broader trend toward catheter-enabled regional therapy (96). As previously mentioned, focused ultrasound sonication in adjunct with MRI monitoring has the capability to transiently increase BBB permeability, thus improving chemotherapeutic delivery (39). Other emerging technologies, including Second Window Indocyanine Green (SWIG) fluorescence guidance, are primarily neurosurgical and radiation oncology modalities but reflect the same image-guided, precision-based principles that underpin interventional radiology in the management of brain metastases, enhancing local control and procedural accuracy. SWIG leverages near-infrared (NIR) fluorescence imaging following preoperative administration of indocyanine green (ICG), which preferentially accumulates in tumor tissue due to blood–brain barrier disruption. Under NIR visualization, metastatic lesions fluoresce intraoperatively, allowing surgeons to better delineate tumor margins in real time, even through overlying normal brain parenchyma. This visualization thereby improves the extent of resection while minimizing injury to eloquent cortex (97, 98). Together, several of these innovations reflect the evolving collaboration between interventional and surgical disciplines to optimize local tumor control and functional outcomes in patients with brain metastases. However, these approaches should not yet be considered established alternatives to surgery, stereotactic radiosurgery, or systemic therapy and currently should be reserved for carefully selected patients enrolled in structured protocols or clinical trials.

#### Evidence, limitations, and patient selection

Preoperative embolization is a selective adjunct for angiographically hypervascular lesions with safely accessible arterial supply, rather than a routine procedure for all metastases. LITT is used mainly for selected recurrent lesions or radiation necrosis, particularly when open surgery is undesirable, but prospective randomized data are limited and lesions larger than approximately 3 cm may be difficult to ablate completely. Risks include edema, hemorrhage, neurologic injury, and nontarget embolization, and treatment decisions should incorporate lesion number, size, location, systemic disease status, prior radiation, symptoms, and expected survival.

#### Future directions

Future studies should identify imaging biomarkers that more reliably distinguish tumor recurrence from treatment-related changes and determine which patients are most likely to benefit from ablation, fluorescence-guided surgery, embolization, or regional drug-delivery approaches.

### Primary central nervous system lymphoma (PCNSL)

#### Conventional imaging

**Figure 5:**
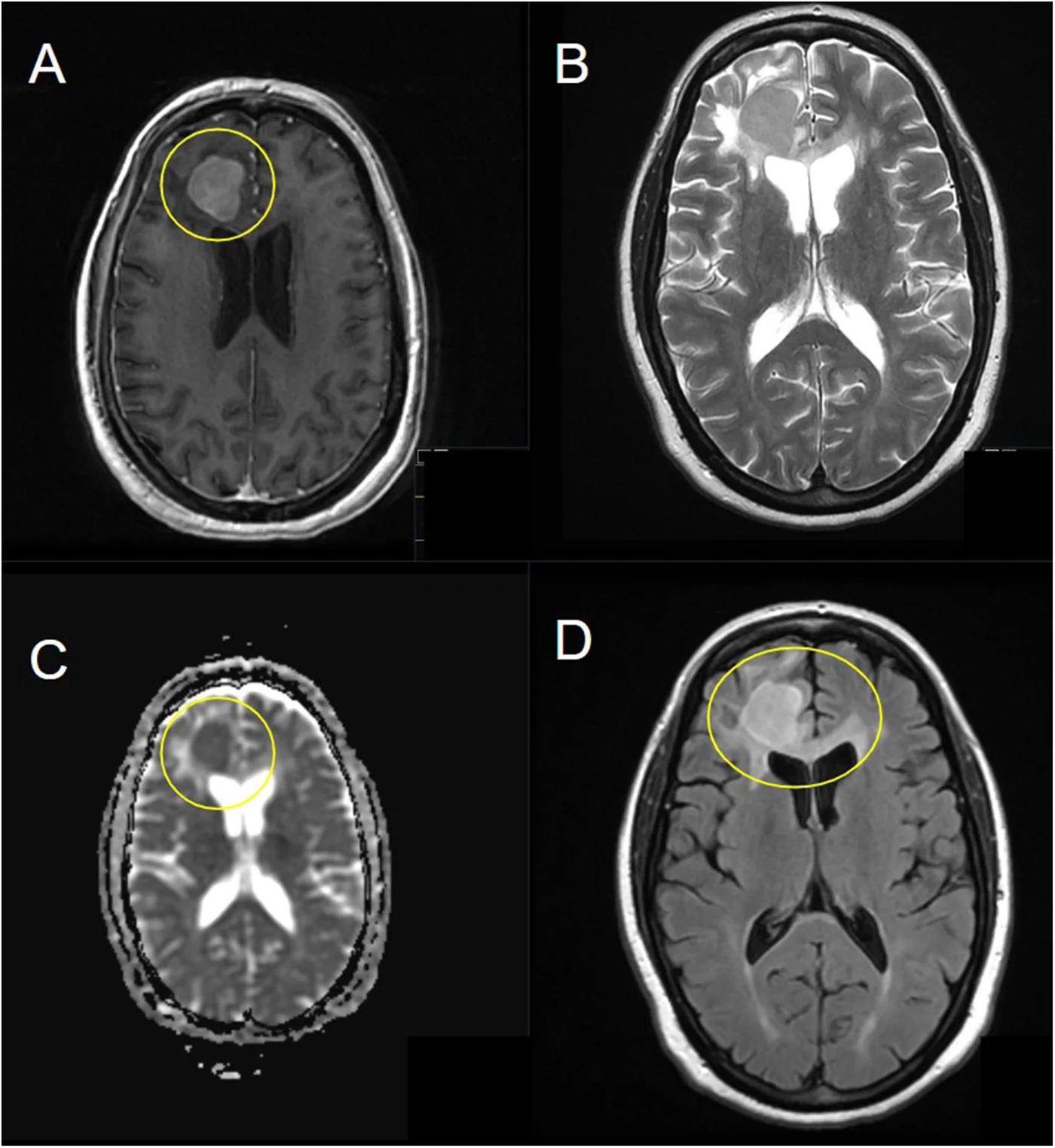
Primary central nervous system lymphoma. **(A)** Axial T1-weighted post-contrast image demonstrates a homogeneously enhancing solid lesion in the right frontal lobe extending into the genu of the corpus callosum without gross internal necrosis (yellow circle). **(B)** Axial T2-weighted image shows an intermediate to hypointense mass. Hyperintense signal extends along the genu to the contralateral side, likely representing reactive edema. Mild to moderate surrounding vasogenic edema is present without midline shift or herniation. **(C)** Apparent diffusion coefficient map demonstrates reduced ADC values within the lesion (yellow circle), suggesting high cellularity and supporting a higher-grade neoplasm. **(D)** Axial FLAIR image demonstrates a homogeneously hyperintense lesion with edema extending across the genu of the corpus callosum (yellow circle) and associated perilesional edema.

PCNSL is an aggressive extranodal non-Hodgkin lymphoma arising in the brain, spinal cord, leptomeninges, or eyes. Although immunosuppression is an important risk factor, most contemporary cases occur in immunocompetent individuals (99)., Lesions involve the deep periventricular white matter, the corpus callosum, basal ganglia, and thalamus, often surrounding the ventricular system (100). On MRI, T2-weighted images display tumors as typically iso- to hypointense relative to gray matter, with intense, homogeneous contrast enhancement (Figure 5) (101, 102). Moreover, lesions show marked restricted diffusion on diffusion-weighted imaging due to high cellularity and limited extracellular space. Several important imaging pitfalls should be acknowledged. Corticosteroid administration prior to imaging can facilitate rapid lesion regression, and consequently, enhancement loss. This phenomenon is labeled as the “vanishing tumor,” and can delay diagnosis (103). Additionally, PCNSL can be a mimicker of imaging characteristics, often overlapping with features seen in high-grade glioma, metastasis, or tumefactive demyelination, particularly in atypical presentations (104).

#### Advanced and molecular imaging

To aid with differentiation, advanced imaging can provide adjunctive diagnostic information. On advanced imaging, perfusion MRI demonstrates low relative cerebral blood volume, reflecting minimal neovascularity compared with other tumors such as high-grade gliomas, while MR spectroscopy possesses elevated choline with prominent lipid peaks, corresponding to high membrane turnover and cellular necrosis (101). Moreover, FDG-PET complements imaging by highlighting intense hypermetabolism and is essential for excluding systemic lymphoma (105).

Advanced MRI techniques such as arterial spin labeling (ASL) provide assessment of tumor perfusion, and demonstrate relatively low perfusion in PCNSL compared to other malignancies such as high-grade gliomas (106, 107). Amide proton transfer (APT) imaging and other chemical exchange saturation transfer techniques are emerging tools that characterize tumor cellularity and protein content, aiding in differentiation of PCNSL from mimickers (108, 109). Furthermore, quantitative diffusion techniques, including diffusion kurtosis imaging and histogram-based ADC analysis, are increasingly being integrated to improve lesion characterization and risk stratification in PCNSL (110–113). These quantitative techniques remain adjunctive, as performance varies with acquisition, region-of-interest selection, corticosteroid exposure, and atypical tumor phenotype.

#### Image-guided procedures

The principal image-guided procedural role in PCNSL is neurosurgery-led stereotactic biopsy to establish an accurate diagnosis, as confirming PCNSL is critical for prognosis and treatment planning, given its high sensitivity to methotrexate-based systemic chemotherapy (114). Debulking procedures are generally avoided because PCNSL is often multifocal, diffusely infiltrative, and located within deep brain structures (114). Biopsy is typically required for confirmation of PCNSL, due to other enhancing intracranial mimickers on imaging. Neurosurgery teams perform stereotactic needle biopsy, either with frame-based or frameless neuronavigation. This is the preferred diagnostic procedure because it permits targeted tissue sampling with relatively low morbidity when an appropriate trajectory is selected. Pre-procedural imaging and planning with neuroradiologists is highly critical in order to find the safest trajectory and avoid eloquent brain structures or major blood vessels (115). MRI T1 sequences for pre-procedural planning are most useful in identifying the most solid, viable portions of tumor that would provide the best tissue sample. Contrast CT is adequate when MRI is contraindicated. Once the stereotactic system is registered to the patient's imaging, a small burr hole is created to advance the biopsy needle through the planned trajectory and obtain tissue samples, often with intra-operative frozen section analysis to confirm sample adequacy (116). As aforementioned, steroids should be avoided prior to procedure due to their promotion of tumor shrinkage, which in turn would cause difficulty in biopsying. If steroids are administered beforehand and the biopsy is non-diagnostic, repeat biopsy is recommended once steroids have been tapered off (116).

In selected centers, iMRI may also support stereotactic biopsy by confirming the target, trajectory, and location of tissue sampling. This may be particularly useful for small or deep lesions and in suspected PCNSL, where prior corticosteroid exposure or intratumoral heterogeneity can reduce diagnostic yield. A recent retrospective study of CNS lymphoma biopsy found that iMRI provided useful information regarding target sampling and anticipated diagnostic adequacy (117). Since the available evidence is retrospective and center dependent, iMRI should be viewed as an adjunct to careful trajectory planning, tissue handling, and intraoperative pathology rather than act as a substitute.

Despite the improvement in treatment and prognosis of PCNSL over the last several decades with systemic chemotherapy, the 5-year survival rate is approximately only 30% to 40%, highlighting the need for new therapeutic approaches to improve patient survival (118). Endovascular intra-arterial chemotherapy with osmotic blood-brain barrier disruption remains an investigational treatment strategy. In a 2023 ASCO meeting abstract describing a multi-institutional, single-arm phase II study, this approach (with methotrexate, rituximab, and carboplatin as the intra-arterial chemotherapy) achieved an 80% radiographic complete response rate, with a median progression-free survival of 55 months and overall survival of 83 months, though with a small sample size of twenty patients (119). The reported outcomes were encouraging relative to historical systemic-therapy cohorts, but fascinatingly were achieved without any whole brain radiation or autologous stem cell transplantation. It is worth noting that grade 3-4 neurologic or hematologic complications were relatively common, but were comparable to the rates of neurologic or hematologic side effects in standard systemic chemotherapy regimens (119). However, as these results are available only as a conference abstract and were derived from a small, nonrandomized cohort, these outcomes should be considered preliminary. In a 2025 large single-center cohort of 642 patients with brain malignancies, including PCNSL, cerebral intra-arterial chemotherapy was shown to be generally safe, with a low symptomatic complication rate of 0.9% per treatment, consisting of primarily ischemic strokes (120). The NCCN guidelines currently recognize intra-arterial chemotherapy with blood-brain barrier disruption as investigational but promising, particularly for patients with refractory disease or contraindications to systemic chemotherapy, underscoring the importance of developing novel regional therapeutic strategies for malignancies traditionally managed with systemic treatment (114). To note, the available evidence comes from small, nonrandomized studies, and the risk of neurologic and hematologic toxicity makes it currently difficult to conclude that this approach is comparable to standard systemic therapy.

#### Evidence, limitations, and patient selection

Conventional MRI and diffusion imaging are established for lesion detection and biopsy planning, but tissue diagnosis remains necessary in most patients. Stereotactic biopsy is established, whereas iMRI is an adjunct available in selected centers. Intra-arterial chemotherapy and blood-brain barrier disruption remain investigational and should generally be limited to specialized programs or trials. Biopsy planning should consider whether the lesion can be safely accessed, whether prior corticosteroid treatment may have reduced diagnostic yield, and whether anticoagulation or immunosuppression increases procedural risk. Imaging should also be used to target viable, representative tumor while avoiding necrotic tissue.

#### Future directions

Future studies should prospectively evaluate whether intraoperative imaging improves first-biopsy diagnostic yield and whether regional treatment strategies provide longer-lasting disease control over current systemic regimens without causing excessive neurotoxicity.

### Meningioma

#### Conventional imaging

**Figure 6:**
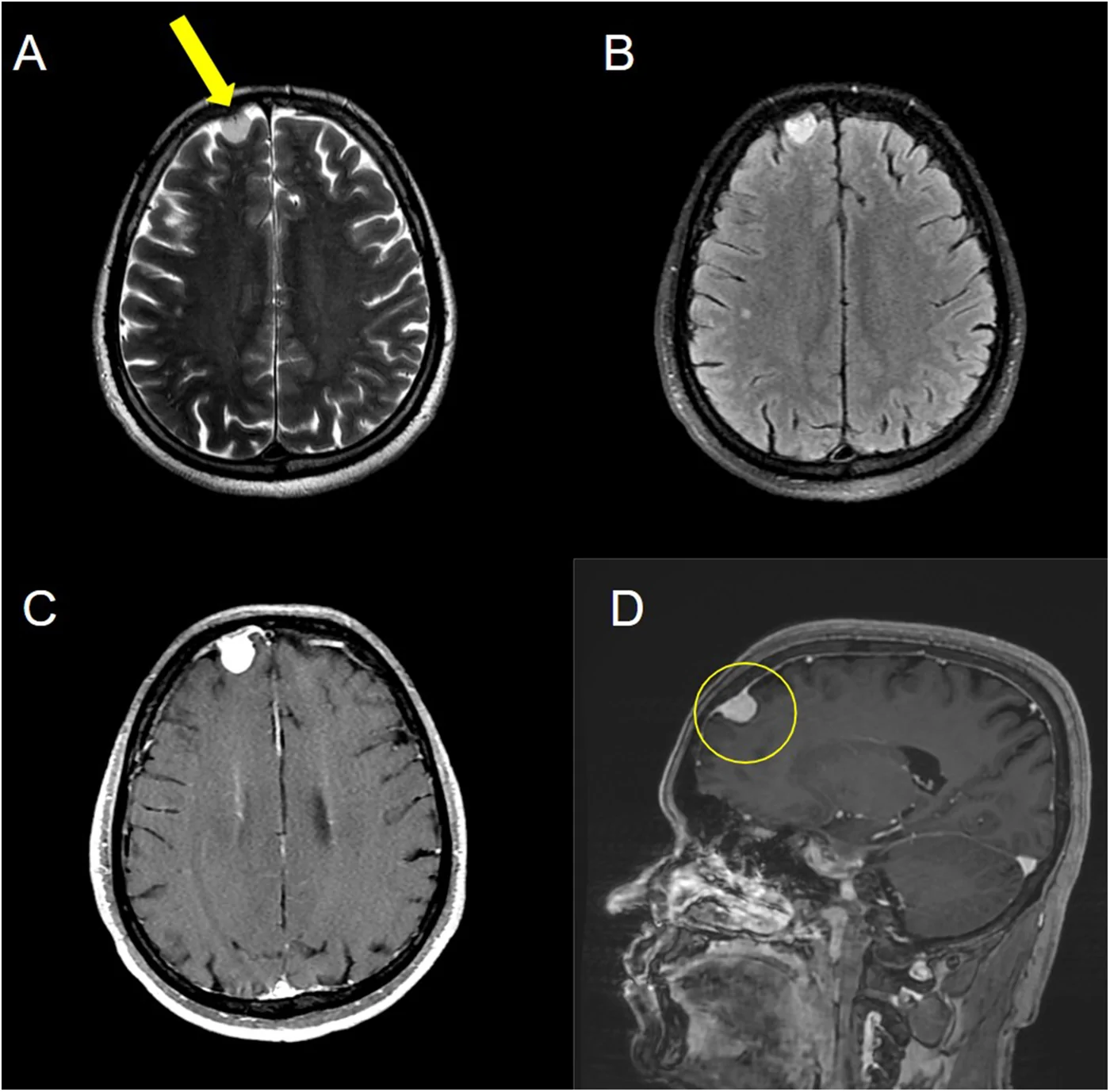
Meningioma. **(A)** Axial T2-weighted image shows a well-circumscribed, solid extra-axial hyperintense lesion in the right anterior frontal region (yellow arrow) without significant surrounding vasogenic edema or mass effect. **(B)** Axial FLAIR image demonstrates the lesion to be hyperintense relative to adjacent brain parenchyma. **(C)** Axial T1-weighted post-contrast image reveals homogeneous solid enhancement with a characteristic dural tail extending along the adjacent dura mater. **(D)** Sagittal T1-weighted post-contrast image confirms avid homogeneous enhancement and the presence of a dural tail (yellow circle), consistent with a dural-based extra-axial mass such as a meningioma.

Meningiomas are the most common primary intracranial tumor (121). The 2021 WHO classification grades meningiomas as CNS WHO grades 1–3 using both histologic and molecular criteria. A TERT promoter mutation or homozygous deletion of CDKN2A/B is sufficient for designation as CNS WHO grade 3, even without otherwise anaplastic histologic features (122). MRI is the gold standard for diagnosing meningioma. Typical WHO grade 1 meningiomas are benign, extra-axial, and dural-based masses arising from the arachnoid cap cells of the meninges. On MRI, meningiomas present as variable T1 hypointense to isointense and T2 isointense to hyperintense extraaxial lesions with typical homogeneous enhancement on post contrast sequences images (121) (Figure 6). An associated enhancing “dural tail” is contiguous with the tumor is often seen (123).

Non-contrast CT scans may be used to detect calcification and bone involvement when diagnosing meningiomas, especially skull-base lesions (124). Typical diagnostic features on CT include a sharply circumscribed lobular mass with a broad-based dural attachment. Meningiomas may appear as homogenous and hyperdense extra-axial masses with 15%–20% of cases exhibiting a speckled hyperdense appearance reflecting calcification (124). CT can also assist with detecting bony changes that are associated with meningiomas, such as hyperostosis, osteolysis, and pneumosinus dilatans. Hyperostosis, the most common osseous finding, appears as focal skull thickening and is frequently associated with otherwise typical, often grade 1 meningiomas (25%–49%) (124). Due to meningioma's hypervascular nature, MR angiography can visualize enhancing vessels within and around the periphery of lesions (124). Meningiomas exhibit a prominent tumor blush with delayed washout, while also impeding on adjacent vessels such as dural venous sinuses (124). Thus, surgical planning for resection often utilizes angiography to help identify potential preoperative embolization to reduce hemorrhage during excision. In contrast, higher-grade meningiomas (WHO grade 2 and 3) may demonstrate more heterogeneous enhancement, irregular or lobulated margins, cystic or necrotic change, greater peritumoral edema, and more aggressive local invasion than typical grade 1 lesions (125).

#### Advanced and molecular imaging

Conventional imaging plays a primary role in detecting meningiomas. Newer methods move beyond this to characterize tumors. With respect to advanced imaging, Combined MR diffusion tensor imaging parameters have shown promise in differentiating high-grade from low-grade meningiomas, a distinction that remains limited on conventional MRI alone (126). Arterial spin labeling and dynamic susceptibility contrast (DSC) perfusion imaging provide quantitative assessment of tumor vascularity and hemodynamics, and thus characterize tumor aggressiveness (127, 128). Susceptibility-weighted imaging (SWI) can help detect intratumoral calcification and hemorrhage, further assisting with tumor characterization (129). Additionally, molecular imaging techniques such as somatostatin receptor PET imaging (e.g., Ga-68 DOTATATE PET) emerged as a sensitive adjunct to conventional imaging (130, 131). More recently, the 2024 joint EANM/EANO/RANO/SNMMI practice guideline identified additional clinical applications, including distinguishing meningioma from non-meningioma tissue, defining tumor extent and osseous involvement for surgical or radiation treatment planning, detecting residual tumor after surgery, and differentiating recurrent disease from treatment-related changes or scar tissue (132). However, its use remains selective and complementary to conventional MRI because availability, reimbursement, and standardized interpretation vary across institutions. AI models have shown preliminary performance in meningioma tumor segmentation, pathological prediction, and prognostication, but routine use is limited by dataset heterogeneity and insufficient prospective external validation (133).

#### Image-guided procedures

Endovascular neurointervention provides a selective adjunctive role in the treatment of meningiomas through pre-operative embolization. The first line curative treatment for meningioma is surgical resection; however, the highly vascular nature of meningiomas make this surgery prone to significant intraoperative bleeding. However, not all meningiomas are good candidates for preoperative embolization. This decision is determined based on multiple factors such as tumor vascularity, location, and size (134). Meningiomas that are supplied by branches of the external carotid artery, most commonly the middle meningeal artery and occasionally the ascending pharyngeal artery, are ideal candidates. This is because they can be safely accessed by microcatheter and embolized without sacrificing critical brain structures. Parasagittal, falcine, and convexity meningiomas are most often supplied by these external carotid artery branches, particularly the middle meningeal artery. On the other hand, meningiomas that are supplied by branches of the internal carotid artery, such as pial arteries or the ophthalmic artery, are poor candidates. These arteries are difficult to access and their embolization is associated with a much higher rate of neurologic complications, as they supply much more eloquent brain structures (135–137). Moreover, embolization of the external carotid artery feeders tends to result in a more complete devascularization of the tumor, translating to a greater reduction in intra-operative blood loss during resection. Embolization has also been shown to increase the safety and completeness of resection by softening the tumor, allowing better visibility, and providing control of surgically inaccessible feeding arteries (138, 139). A recent molecular analysis reported associations between embolization, altered tumor-cell pathways, and recurrence-free survival in atypical (WHO grade II) meningioma (140). This finding requires prospective confirmation before an oncologic benefit can be inferred.

New advances in meningioma embolization are aimed at maximizing tumor devascularization while minimizing complications. Utilization of modern liquid embolic agents, such as ethylene-vinyl alcohol copolymer (Onyx) and precipitating hydrophobic injectable liquid (PHIL), promote deeper intratumoral penetration and increased durability in comparison with traditional particulate embolization (141, 142). Furthermore, developments in superselective microcatheter design, flow-directed systems, and cone-beam CT angiography have improved safely targeting tiny distal tumor-specific vessels (127, 143–145). Additionally, emerging quantitative assessment and grading approaches for tumor embolization, incorporating volumetric MRI measurements and angiographic devascularization scores, are being investigated to better characterize embolization effectiveness, inform surgical complexity, and predict intraoperative outcomes (146).

#### Evidence, limitations, and patient selection

Preoperative embolization is a selectively established adjunct rather than a routine component of meningioma surgery. The strongest candidates are large, hypervascular tumors with dominant, safely accessible external carotid artery supply and anticipated operative blood loss. Evidence for reduced blood loss or reduced operative difficulty is largely retrospective and heterogeneous, while risks include ischemic injury, cranial neuropathy, visual loss, hemorrhage, tumor swelling, and nontarget embolization. Internal carotid, ophthalmic, or substantial pial supply may increase procedural risk.

#### Future directions

Looking forward, the role of interventional and image-guided technologies in meningioma management is expected to expand. AI-driven predictive modeling is being developed to create patient-specific “digital twin” models to optimize procedural and surgical planning (147). Emerging minimally invasive technologies such as robotic-assisted endovascular navigation, MR-guided focused ultrasound for noninvasive tumor modulation, and next-generation laser interstitial thermal therapy platforms with real-time thermal feedback algorithms are being investigated as adjuncts or alternatives to open resection of CNS tumors, such as meningioma (148, 149). These approaches remain investigational and require prospective evaluation to determine their safety, appropriate patient-selection criteria, procedural benefits, and effects on tumor control and patient outcomes.

### Summary

**Figure 7:**
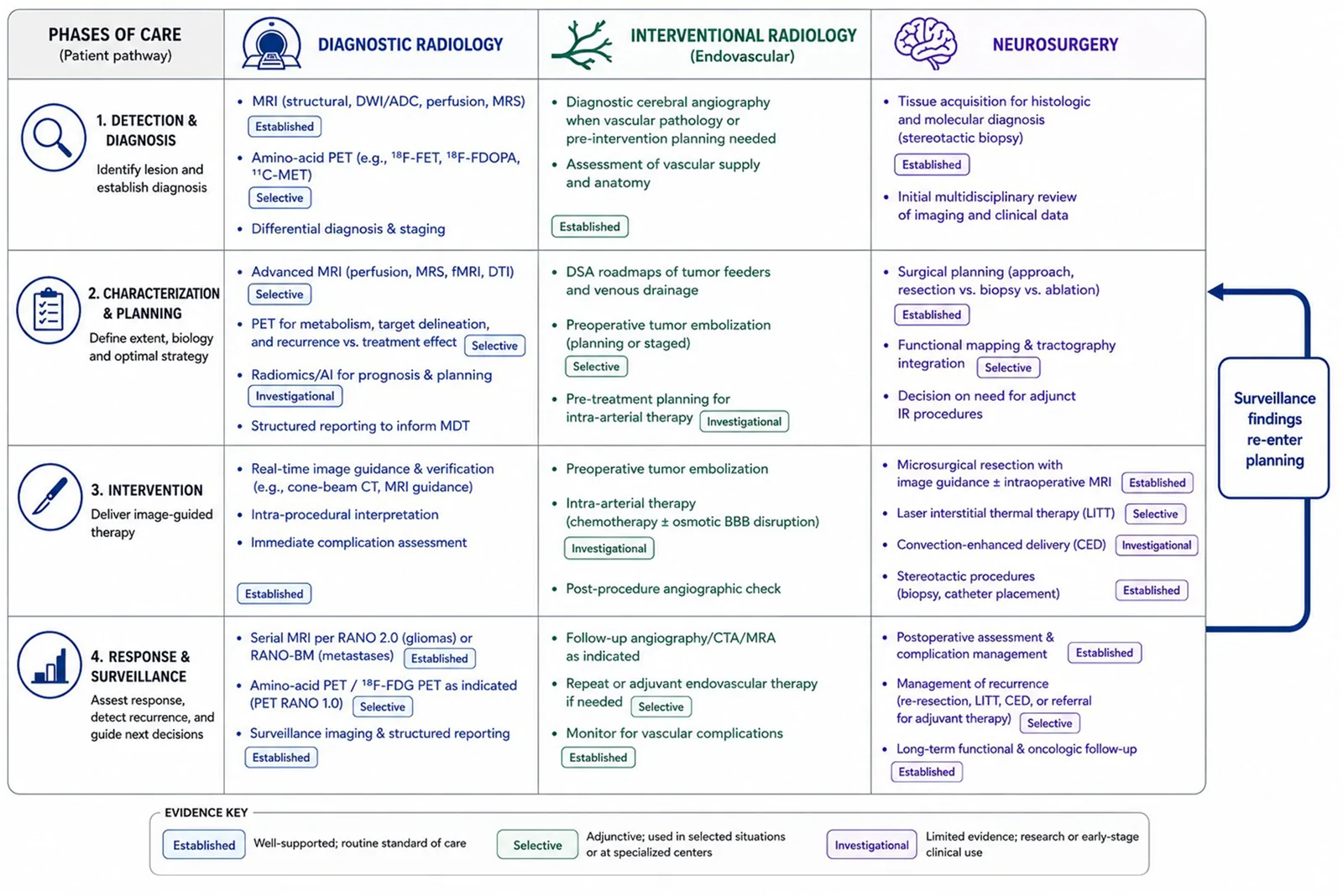
Multidisciplinary imaging and image-guided workflow in neuro-oncologic care. The matrix summarizes the complementary contributions of diagnostic radiology, endovascular interventional radiology, and neurosurgery across detection and diagnosis, characterization and treatment planning, intervention, and response assessment and surveillance. The return arrow indicates that surveillance findings may prompt renewed multidisciplinary evaluation and re-entry into treatment planning. DWI, diffusion-weighted imaging; ADC, apparent diffusion coefficient; MRS, magnetic resonance spectroscopy; fMRI, functional magnetic resonance imaging; DTI, diffusion tensor imaging; DSA, digital subtraction angiography; LITT, laser interstitial thermal therapy; CED, convection-enhanced delivery; CTA, computed tomography angiography; MRA, magnetic resonance angiography; RANO, Response Assessment in Neuro-Oncology; RANO-BM, Response Assessment in Neuro-Oncology Brain Metastases.

The principal diagnostic imaging modalities and image-guided interventions discussed across the five tumor types are summarized in Tables 1, 2, respectively. Figure 7 complements these tabular summaries by illustrating how diagnostic radiology, endovascular interventional radiology, and neurosurgery contribute across the longitudinal care pathway, with surveillance findings feeding back into multidisciplinary treatment planning.

**Table 1:** Diagnostic imaging modalities across Major brain tumor types: clinical questions, evidence Status, and limitations .

Tumor type	Imaging modality	Principal clinical question addressed	Current evidence and clinical role	Key limitations
Glioblastoma	Contrast-enhanced MRI with DWI and ADC mapping	What is the tumor location and extent, where is viable cellular tumor located, and how has the lesion changed following treatment?	Established clinical standard for diagnosis, surgical and biopsy planning, postoperative assessment, and surveillance. RANO 2.0 provides the principal MRI- and clinically based framework for treatment-response assessment in adult diffuse gliomas.	Enhancement and T2/FLAIR abnormalities are nonspecific and may reflect tumor, edema, pseudoprogression, radiation injury, or other treatment-related changes.
Glioblastoma	Perfusion MRI and MR spectroscopy	Is an abnormality likely to represent viable tumor, and does an equivocal post-treatment lesion represent progression or treatment effect?	Selective clinical adjunct. Elevated perfusion and altered metabolite patterns may support tumor characterization, biopsy targeting, prognostication, and differentiation of progression from treatment-related change.	Variability in acquisition, leakage correction, postprocessing, voxel placement, and interpretive thresholds; no individual parameter is definitive.
Glioblastoma and other adult diffuse gliomas	Amino-acid PET with ^18F-FET, ^18F-FDOPA, or ^11C-MET	What is the metabolically active tumor extent, where should biopsy or local treatment be targeted, and does an abnormality represent recurrence or treatment effect?	Guideline-supported selective adjunct. Amino-acid PET may complement MRI for tumor delineation, treatment targeting, and evaluation of suspected recurrence. PET RANO 1.0 provides complementary metabolic response criteria.	Limited tracer availability, radiation exposure, heterogeneous acquisition and interpretation methods, and variable reimbursement; it does not replace MRI or tissue diagnosis.
Low-grade glioma	Conventional and serial MRI	What is the lesion extent and growth trajectory, and are there imaging findings suggesting malignant progression?	Established clinical standard for diagnosis, operative planning, surveillance, and detection of interval growth or transformation.	Nonenhancing infiltrative margins may be difficult to define, and conventional imaging cannot establish the complete integrated molecular diagnosis.
Low-grade glioma	Functional MRI, DTI, and tractography	What is the relationship between the tumor, eloquent cortex, and major white-matter pathways?	Established or selectively used surgical-planning adjunct at experienced centers. These techniques may influence operative approach, biopsy trajectory, and anticipated functional risk.	Functional MRI may be affected by task performance and neurovascular uncoupling. Tractography depends on acquisition and modeling assumptions and does not directly demonstrate functional integrity.
Low-grade glioma	2-hydroxyglutarate MR spectroscopy and MRI-based molecular prediction	Can IDH status, 1p/19q codeletion, or tumor grade be estimated noninvasively before surgery?	Specialized or investigational adjunct. These approaches may support preoperative counseling, biopsy planning, and research stratification.	Limited availability, scanner and protocol dependence, small or imbalanced datasets, model overfitting, and limited prospective external validation. These techniques cannot replace tissue-based molecular classification.
Brain metastases	Contrast-enhanced MRI	How many intracranial lesions are present, where are they located, and how have they responded to treatment?	Established clinical standard for detection, stereotactic-radiosurgery or surgical planning, and surveillance. RANO-BM provides a standardized MRI- and clinically based response framework.	Very small lesions may be difficult to measure reliably, and post-treatment enhancement may represent either recurrence or radiation-related change.
Brain metastases	Perfusion MRI and MR spectroscopy	Does an enlarging treated lesion represent recurrent metastasis or radiation necrosis?	Selective clinical adjunct when conventional MRI is inconclusive. Multiparametric interpretation may improve confidence in distinguishing recurrence from treatment effect.	Considerable overlap between entities, susceptibility artifacts, protocol dependence, and lack of a universally accepted diagnostic threshold.
Brain metastases	Amino-acid PET with ^18F-FET, ^18F-FDOPA, or ^11C-MET	Does an equivocal post-treatment lesion represent recurrent tumor or radiation-related change?	Guideline-supported adjunct to MRI in selected patients. It may be particularly useful when structural and perfusion imaging remain inconclusive.	Limited tracer availability, variable interpretation thresholds, and incomplete prospective validation across primary cancer types and treatment settings.
PCNSL	Contrast-enhanced MRI with DWI and ADC mapping	Does the lesion have a characteristic PCNSL imaging phenotype, and where is the safest viable biopsy target?	Established clinical standard for lesion detection, differential diagnosis, and biopsy planning. Marked diffusion restriction may support the diagnosis, although tissue confirmation is generally required.	Corticosteroids may reduce enhancement and diagnostic yield. Atypical PCNSL may mimic glioblastoma, metastasis, demyelination, or infection.
PCNSL	Perfusion MRI, ASL, and MR spectroscopy	Can PCNSL be differentiated from glioblastoma or other enhancing intracranial lesions?	Selective clinical adjunct. Relatively low perfusion and characteristic metabolic features may support differentiation from more vascular tumors.	Acquisition and region-of-interest dependence, overlapping findings in atypical lesions, corticosteroid effects, and variable thresholds.
PCNSL	^18F-FDG PET/CT	Is systemic lymphoma present, and can metabolic imaging support staging or prognostication?	Guideline-supported staging adjunct, particularly for identifying extracranial disease. Intracranial metabolic findings complement MRI.	High physiologic cerebral glucose uptake may reduce intracranial lesion conspicuity, and inflammatory or infectious abnormalities may also be FDG-avid.
PCNSL	APT imaging and advanced quantitative diffusion	Can tumor cellularity, protein content, or microstructural features improve differentiation from tumor mimics?	Emerging or investigational. Initial studies report promising diagnostic performance, but routine clinical roles have not been established.	Limited multicenter standardization, acquisition dependence, small cohorts, and insufficient prospective validation.
Meningioma	Contrast-enhanced MRI and CT	Is the lesion consistent with meningioma, what is its intracranial extent, and is there calcification or osseous involvement?	Established clinical standard. MRI is the primary modality, while CT provides complementary assessment of calcification, hyperostosis, osteolysis, and skull-base anatomy.	Imaging cannot reliably determine histologic or molecular grade, and other dural-based lesions may have similar appearances.
Meningioma	Diffusion, perfusion/ASL, and susceptibility-weighted imaging	Can vascularity, hemorrhage, calcification, or the likelihood of higher-grade disease be characterized?	Selective clinical adjunct for tumor characterization and operative or embolization planning.	Substantial overlap among grades, scanner and protocol dependence, and inability to replace tissue-based grading.
Meningioma	Somatostatin-receptor PET, including ^68Ga-DOTATATE PET	Is indeterminate tissue residual or recurrent meningioma, is bone involved, and what tissue should be included in a radiation target?	Guideline-supported selective adjunct for tumor delineation, osseous involvement, postoperative residual disease, suspected recurrence, and selected radiation-planning questions.	Limited availability, variable reimbursement, radiation exposure, and incomplete harmonization of acquisition and interpretation.
Across tumor types	Artificial intelligence and radiomics	Can tumors be detected or segmented automatically, molecular features predicted, prognosis estimated, or treatment response assessed quantitatively?	Investigational for most applications, despite promising retrospective diagnostic and prognostic performance.	Dataset shift, preprocessing and segmentation variability, model overfitting, limited prospective external validation, interpretability, and uncertain effects on patient outcomes.

Evidence status represents a qualitative narrative synthesis rather than a formal GRADE assessment. “Established clinical standard” indicates routine use supported by guidelines or broad clinical consensus; “selective adjunct” indicates use for defined clinical questions or at specialized centers; and “investigational” indicates insufficient evidence for routine clinical implementation.

**Table 2:** Selected image-guided neuro-oncologic interventions: indications, performing specialty, evidence Status, guideline position, and limitations .

Tumor or clinical setting	Intervention	Principal indication	Primary performing specialty	Current evidence status	Guideline or consensus position	Key limitations
Glioma surgery	Intraoperative MRI	Update neuronavigation after brain shift, identify residual tumor, and guide additional safe resection	Neurosurgery-led, with neuroradiology and intraoperative imaging support	Established adjunct at selected centers. Evidence supports improved identification of residual tumor and may increase extent of resection; effects on survival and cost-effectiveness remain less certain.	No major guideline requires routine use. It may be used at appropriately equipped centers to support maximal safe resection.	High capital and maintenance costs, limited availability, additional operative time, anesthesia and workflow complexity, MRI-compatible equipment, and artifacts.
PCNSL or other selected stereotactic biopsies	Intraoperative MRI	Confirm biopsy target, trajectory, and sampling location	Neurosurgery-led, with neuroradiology support	Selective adjunct supported mainly by retrospective evidence.	Guidelines recommend stereotactic tissue acquisition for PCNSL but do not require intraoperative MRI. It should be presented as an optional procedural adjunct.	Cost, availability, added workflow complexity, and uncertain incremental improvement in first-biopsy diagnostic yield.
Glioblastoma or low-grade glioma	MR-guided laser interstitial thermal therapy	Minimally invasive cytoreduction of selected deep, recurrent, or surgically high-risk lesions	Neurosurgery-led	Selective clinical use. Evidence consists predominantly of retrospective cohorts, institutional series, pilot studies, and heterogeneous systematic reviews.	NCCN permits consideration of LITT in selected patients who are poor candidates for craniotomy or conventional resection. It is not a replacement for maximal safe resection in otherwise appropriate surgical candidates.	Limited randomized comparative evidence, lesion-size constraints, edema, thermal injury, heat-sink effects, and proximity to eloquent structures.
Brain metastasis or radiation necrosis	MR-guided laser interstitial thermal therapy	Ablation of selected recurrent metastases or symptomatic radiation necrosis when open surgery is undesirable	Neurosurgery-led	Selective clinical use, with limited prospective comparative evidence.	ASCO–SNO–ASTRO states that no recommendation can be made for or against LITT because available evidence is insufficient.	Incomplete ablation of large or irregular lesions, postoperative edema, corticosteroid requirements, hemorrhage, and neurologic injury.
Glioblastoma	Convection-enhanced delivery	Direct intraparenchymal administration of chemotherapy, immunotoxins, viral vectors, or nanoparticle-based therapies	Neurosurgery-led, with neuro-oncology, neuroradiology, pharmacy, and engineering support	Investigational. Evidence consists mainly of early-phase trials and small nonrandomized cohorts.	No established routine role in major glioma guidelines; generally limited to clinical trials or specialized research programs.	Heterogeneous intratumoral distribution, reflux, catheter-position dependence, tissue-pressure variability, and uncertain durable clinical benefit.
High-grade glioma or glioblastoma	Microbubble-enhanced focused-ultrasound blood–brain barrier opening	Transient localized BBB opening to improve drug delivery	Multidisciplinary, involving focused-ultrasound specialists, neurosurgery, neuroradiology, and neuro-oncology	Early-phase investigational. Clinical studies support feasibility and short-term safety, but definitive therapeutic efficacy has not been established.	No established routine guideline role; use should remain within clinical trials or specialized investigational protocols.	Skull-dependent acoustic transmission, specialized equipment, repeated-treatment logistics, imaging and dosimetry requirements, and lack of phase III efficacy evidence.
Glioblastoma	Superselective intra-arterial therapy with or without osmotic BBB disruption	Increase regional drug delivery in selected newly diagnosed, recurrent, or otherwise difficult-to-treat tumors	Endovascular neurointervention	Investigational. Evidence is dominated by feasibility studies, nonrandomized series, computer modeling, and ongoing early-phase trials.	No established standard role in major glioblastoma guidelines; clinical-trial participation is preferred.	Heterogeneous agents and protocols, ischemic or hemorrhagic complications, catheter-related risk, and uncertain survival benefit.
Glioblastoma	Preoperative embolization	Devascularize an unusually hypervascular tumor with dominant, safely accessible feeding arteries before resection	Endovascular neurointervention	Highly selective and investigational, supported by very small institutional series.	No routine role in glioblastoma management guidelines. Consideration should be limited to unusual cases with favorable angiographic anatomy.	Most glioblastomas are not sufficiently hypervascular; risks include ischemia, hemorrhage, nontarget embolization, and limited generalizability.
PCNSL	Stereotactic biopsy	Obtain tissue for histologic and molecular diagnosis	Neurosurgery-led, with neuroradiology and neuropathology support	Established diagnostic procedure.	EHA–ESMO identifies histopathologic examination of tissue obtained by stereotactic biopsy as the diagnostic standard in most patients.	Corticosteroid-related lesion regression, sampling error, hemorrhage, nondiagnostic tissue, and deep or eloquent lesion location.
PCNSL	Intra-arterial chemotherapy with osmotic BBB disruption	Regional administration of methotrexate-based or combination therapy in selected specialized programs	Endovascular neurointervention, with hematology and neuro-oncology support	Investigational. Evidence is based on small nonrandomized cohorts, retrospective safety analyses, and conference-abstract data.	Not established as standard first-line or salvage therapy in EHA–ESMO guidance; systemic high-dose methotrexate-based therapy remains standard.	Neurologic and hematologic toxicity, ischemic risk, repeated procedures, limited availability, and inability to establish equivalence to systemic therapy.
Hypervascular brain metastasis	Preoperative embolization	Reduce operative bleeding from selected metastases with angiographic tumor blush, particularly from hypervascular primary tumors	Endovascular neurointervention	Highly selective clinical adjunct, supported mainly by small series and extrapolation from tumor-embolization practice.	Not recommended as a routine treatment for brain metastases; use is individualized according to vascular anatomy and surgical goals.	Limited comparative evidence, nontarget embolization, ischemia, visual loss, cranial neuropathy, and usefulness only with safely accessible arterial supply.
Brain-metastasis surgery	Second-window indocyanine-green fluorescence guidance	Improve intraoperative tumor visualization and margin identification	Neurosurgery-led	Emerging or investigational surgical adjunct, supported by small prospective and retrospective cohorts.	No established recommendation in major brain-metastasis guidelines.	Requires preoperative dye administration and specialized near-infrared imaging; limited comparative evidence and uncertain effects on long-term outcomes.
Meningioma	Preoperative embolization	Devascularize a large hypervascular lesion with dominant, safely accessible arterial supply before surgical resection	Endovascular neurointervention	Selective clinical adjunct. Evidence is predominantly retrospective, heterogeneous, and affected by substantial selection bias.	Not recommended as a routine component of all meningioma resections; individualized consideration may be appropriate for selected hypervascular tumors with favorable arterial anatomy.	Ischemic injury, hemorrhage, tumor swelling, cranial neuropathy, visual loss, nontarget embolization, and greater risk with ophthalmic, internal carotid, or substantial pial supply.

Evidence categories represent a qualitative narrative synthesis and are not formal GRADE ratings. Guideline positions summarize the major professional guidance discussed in this review. “No established routine role” indicates that a procedure has not been incorporated into standard care and is generally restricted to selected centers, individualized cases, or clinical investigation.

### Perspectives

Current neuro-oncological focused research can work to expand on the collaboration among diagnostic radiology, endovascular neurointervention, neurosurgery, neuro-oncology, and radiation oncology. Although many institutions already manage brain tumors through highly multidisciplinary collaboration, specialty contributions are often studied and reported as separate components of care rather than as formally evaluated, integrated workflows. Imaging modalities such as MRI and advanced imaging in DR serve well-established roles in diagnosis, procedural planning, and surveillance. However, there remains limited evidence prospectively evaluating how advanced imaging modalities can be incorporated into image–guided procedural protocols, particularly as both endovascular intervention and neurosurgical practices increasingly adopt image-guided management strategies. The 2025 Society of Neurointerventional Surgery report acknowledges the importance of this multidisciplinary model, noting that multidisciplinary collaboration is integral to success (150). To our knowledge, there are currently no published large-scale prospective studies specifically designed to evaluate integrated workflows in brain tumor management. Future studies could investigate the effect of formalized combined workflows and collaboration on several downstream clinical endpoints such as diagnostic yield, complications, local control, and time to next therapy. Other potential explorations include utilizing imaging biomarkers to guide pre-procedural planning.

Moreover, with the rapid evolution of artificial intelligence, there is potential to be had in utilizing these tools to improve workflow and neuro-oncological decision-making, and thus help strengthen coordination between diagnostic imaging and image-guided intervention. For example, multimodal advanced neuroimaging is being combined with radiomic and AI-driven analytical tools for real-time response assessment and procedural planning, particularly for glioblastoma management (151). Another representative clinical study combined radiomic features from MR-based oxygen metabolism and deep convolutional neural networks, showing reliable pre-therapeutic differentiation of glioblastoma and brain metastases in a clinical setting (152). Additionally, Priya et al. offered a radiomics model with three-class classification to differentiate between glioblastoma, metastases, and primary CNS lymphoma with accuracy (153). However, despite offering promise for improving diagnostic accuracy and workflow efficiency, routine adoption of these novel AI tools necessitates more prospective studies to demonstrate procedural benefits and offer seamless integration into current workflows.

Lastly, theranostic approaches that integrate diagnostic and therapeutic modalities are promising. For theranostics to reach clinical maturity in neuro-interventional oncology, research should move beyond isolated single-modality investigations toward coordinated multimodal trials evaluating how diagnostic imaging, image-guided intervention, and systemic therapy can be combined in selected clinical settings. Traditional brain tumor trials have largely treated imaging as a passive monitoring tool rather than an active guide for therapeutic selection and delivery (154). Future clinical trials should therefore prioritize synergistic treatment strategies that integrate theranostic platforms with established modalities such as immunotherapy and radiation therapy, leveraging imaging not only to assess response but to direct and personalize treatment (155). As theranostic workflows evolve, a longitudinal, multidisciplinary model should be encouraged to reflect the evolving growth of image-guided neuro-oncologic care, especially as the incidence and complexity of these neoplasms increase (1).

### Limitations

This narrative review has several limitations. The literature search was not systematic and did not use formal dual-reviewer screening or standardized quality appraisal, creating the possibility of study-selection and publication bias. The available evidence is also heterogeneous and includes professional guidelines, systematic reviews, retrospective cohorts, small single-center series, early-phase trials, conference abstracts, and ongoing trial registrations. Direct comparisons among modalities should therefore be interpreted cautiously and should not be considered formal comparative-effectiveness conclusions. In addition, the rapid development of neuro-oncologic imaging and image-guided interventions may limit the completeness of the review after the final search date. Finally, the review prioritizes a broad clinically oriented synthesis across five major tumor types rather than exhaustive technical discussion of every imaging sequence, molecular tracer, procedural platform, or tumor subtype.

## Conclusion

Closer coordination between advanced diagnostic imaging and image-guided neuro-oncologic interventions marks a crucial step toward a precision-medicine approach for complex brain neoplasms, allowing physicians to provide tailored care pathways that address both the diversity of tumor biology and patient needs (3, 8, 156). Multidisciplinary collaboration among treatment professionals including neuroradiologists, neurointerventionalists, neurosurgeons, and oncologists is needed to maximize diagnostic and therapeutic potential of these technologies (particularly as technological advancements continue to evolve, as demonstrated in the examples discussed in this article) and to ensure positive patient outcomes (150, 157). Although these disciplines increasingly intersect, a validated and broadly applicable longitudinal imaging–intervention workflow has not yet been established. Future prospective studies should determine whether formalized multidisciplinary pathways improve diagnostic yield, treatment selection, procedural safety, and patient outcomes.
