# Correction: Diagnostic imaging and emerging image-guided neurointervention in brain tumors: a narrative review

**DOI:** 10.3389/fradi.2026.1993945

**Published:** 2026-10-27

**Authors:** Zahin Alam, Triston Messer, Anirudh Maddali, Jake Heath, Kush Desai, Srihari Sundararajan, David Monoky, Brij Reddy

**Affiliations:** 1 Department of Radiology, Hackensack Meridian School of Medicine, Nutley, NJ, United States; 2 Department of Interventional Radiology, Robert Wood Johnson Medical School, New Brunswick, NJ, United States; 3 Department of Radiology, Hackensack University Medical Center, Hackensack, NJ, United States; 4 Clearview Radiology PC, Bayside, NY, United States

**Keywords:** brain tumors, diagnostic neuro-imaging, image-guided intervention, image-guided neuro-oncology, interventional neuroradiology, neuro-oncology, neuroradiology, neurosurgery

There was a mistake in the caption of Figure 5 as published. In panel (B), “Axial T2-weighted image shows a hyperintense mass” was incorrect; it should read “Axial T2-weighted image shows an intermediate to hypointense mass”. The corrected caption of Figure 5 appears below.

“Primary central nervous system lymphoma. (A) Axial T1-weighted post-contrast image demonstrates a homogeneously enhancing solid lesion in the right frontal lobe extending into the genu of the corpus callosum without gross internal necrosis (yellow circle). (B) Axial T2-weighted image shows an intermediate to hypointense mass. Hyperintense signal extends along the genu to the contralateral side, likely representing reactive edema. Mild to moderate surrounding vasogenic edema is present without midline shift or herniation. (C) Apparent diffusion coefficient map demonstrates reduced ADC values within the lesion (yellow circle), suggesting high cellularity and supporting a higher-grade neoplasm. (D) Axial FLAIR image demonstrates a homogeneously hyperintense lesion with edema extending across the genu of the corpus callosum (yellow circle) and associated perilesional edema.”

There was a mistake in the caption of Figure 3 as published. The Figure 3 caption incorrectly referred to the annotations in panels (A)–(E) as “yellow arrows”; all instances should read “yellow circles”. The corrected caption of Figure 3 appears below.

“Low-grade glioma. (A) Axial FLAIR demonstrates a hyperintense intraaxial lesion within the high right anterior frontal lobe (yellow circle). (B) Axial DWI reveals no restriction (yellow circle). (C) ADC map shows higher value of the lesion (yellow circle). (D) Axial T1-weighted pre-contrast MRI shows hypointense lesion (yellow circle). (E) Axial T1-weighted post-contrast MRI shows no abnormal enhancement (yellow circle).”

The sentence “According to the 2021 CNS WHO classification, glioblastoma refers specifically to an adult-type diffuse glioma that is IDH-wildtype and classified as CNS WHO grade 4.” has been revised for clarity.

A correction has been made to the section Discussion, Glioblastoma, Conventional imaging, Paragraph 1:

“Glioblastoma refers specifically to an adult, IDH-wildtype, WHO grade 4 neoplasm. IDH-wildtype glioblastomas represent approximately 90% of adult glioblastomas and correspond to the entity historically regarded as “primary” glioblastoma. These tumors exhibit a more aggressive biological behavior and are associated with a poorer prognosis.”

The sentence “Traditionally, it is diagnosed via classic histopathological features, namely necrosis and/or microvascular proliferation, within an IDH-wildtype diffuse astrocytic tumor.” has been revised for clarity.

A correction has been made to the section Discussion, Glioblastoma, Conventional imaging, Paragraph 3:

“Traditionally, tumor diagnosis is achieved with identification of classic histopathological features, namely necrosis and/or microvascular proliferation, within an IDH-wildtype diffuse astrocytic tumor.”

The sentence “Intraoperative MRI provides updated anatomic information that can identify residual tumor, correct neuronavigation, and guide additional resection when this can be performed without unacceptable functional risk.” has been revised.

A correction has been made to the section Discussion, Glioblastoma, Image-guided procedures, Paragraph 4:

“Intraoperative MRI provides updated anatomic information that can identify residual tumor, correct neuronavigation, and guide additional resection. Although iMRI may improve the extent of resection, its effects on long-term survival and cost-effectiveness remain less certain.”

The sentence “Imaging pitfalls include abscesses, tumefactive demyelination, and subacute infarction, all of which may demonstrate enhancing mass-like features and mimic metastatic disease on conventional imaging (82).” has been revised.

A correction has been made to the section Discussion, Metastatic brain disease, Conventional imaging, Paragraph 3:

“In certain cases, diagnostic uncertainty may be introduced due to imaging feature overlap with alternative considerations, including enhancing foci of demyelination, microabscesses, and subacute infarction.”

The sentence “As dural opening, cerebrospinal fluid drainage, edema changes, and tissue removal can produce brain shift, navigation based solely on preoperative imaging may become less accurate as surgery progresses.” has been revised for clarity.

A correction has been made to the section Discussion, Glioblastoma, Image-guided procedures, Paragraph 3:

“During surgery, opening the dura, draining cerebrospinal fluid, changes in edema, and removing tumor tissue can cause the brain to shift from its position on preoperative imaging. As a result, neuronavigation based only on preoperative scans may become progressively less accurate as the operation continues.”

The original version of this article has been updated.

