# Supplementary material for: Diagnostic imaging and emerging image-guided neurointervention in brain tumors: a narrative review
Source: Front Radiol. 2026 Sep 8;6:1914188. doi: 10.3389/fradi.2026.1914188 (PMC13594585; doi:10.3389/fradi.2026.1914188)
Supplement: Supplementary file 1 [file Table1.docx]

**Supplementary Table S1. Literature Search Strategy and Eligibility Framework**

**Part A. Database-specific and supplementary search strategies**

| **Source** | **Search date and coverage** | **Exact strategy or retrieval approach** | **Limits and handling** |
| --- | --- | --- | --- |
| **PubMed/MEDLINE** | Initial search: December 2025 Final update: 31 July 2026 Coverage: database inception–31 July 2026 | (  "Glioblastoma"[Mesh] OR glioblastoma*[Title/Abstract] OR "high-grade glioma*"[Title/Abstract]  OR "low-grade glioma*"[Title/Abstract] OR "IDH-mutant glioma*"[Title/Abstract]  OR oligodendroglioma*[Title/Abstract]  OR "Brain Neoplasms, Secondary"[Mesh] OR "brain metastas*"[Title/Abstract]  OR "intracranial metastas*"[Title/Abstract]  OR "Lymphoma, Primary Central Nervous System"[Mesh]  OR "primary central nervous system lymphoma*"[Title/Abstract] OR PCNSL[Title/Abstract]  OR "Meningioma"[Mesh] OR meningioma*[Title/Abstract] ) AND (  "Magnetic Resonance Imaging"[Mesh] OR MRI[Title/Abstract] OR diffusion[Title/Abstract]  OR DWI[Title/Abstract] OR perfusion[Title/Abstract] OR spectroscopy[Title/Abstract]  OR MRS[Title/Abstract] OR PET[Title/Abstract] OR "positron emission tomography"[Title/Abstract]  OR radiomic*[Title/Abstract] OR radiogenomic*[Title/Abstract]  OR "artificial intelligence"[Title/Abstract] OR "machine learning"[Title/Abstract]  OR "functional MRI"[Title/Abstract] OR fMRI[Title/Abstract]  OR tractography[Title/Abstract] OR "diffusion tensor imaging"[Title/Abstract]  OR DTI[Title/Abstract] OR "intraoperative MRI"[Title/Abstract] OR iMRI[Title/Abstract]  OR "laser interstitial thermal therapy"[Title/Abstract] OR LITT[Title/Abstract]  OR "convection-enhanced delivery"[Title/Abstract] OR CED[Title/Abstract]  OR "focused ultrasound"[Title/Abstract]  OR "blood-brain barrier disruption"[Title/Abstract]  OR "blood brain barrier opening"[Title/Abstract]  OR "intra-arterial"[Title/Abstract] OR embolization[Title/Abstract]  OR embolisation[Title/Abstract] OR "stereotactic biopsy"[Title/Abstract]  OR "image-guided"[Title/Abstract] OR neurointervention*[Title/Abstract] ) AND ("1900/01/01"[Date - Publication] : "2026/07/31"[Date - Publication]) | English-language records. No human-only filter was applied because the review also discusses selected preclinical, in-silico, and early translational evidence. Peer-reviewed full-text publications were prioritized. |
| **Scopus** | Initial search: December 2025 Final update: 31 July 2026 Coverage: database inception–31 July 2026 | TITLE-ABS-KEY(  (  glioblastoma* OR "high-grade glioma*" OR "low-grade glioma*"  OR "IDH-mutant glioma*" OR oligodendroglioma*  OR "brain metastas*" OR "intracranial metastas*"  OR "primary central nervous system lymphoma*" OR PCNSL OR meningioma*  )  AND  (  MRI OR "magnetic resonance imaging" OR diffusion OR DWI OR perfusion  OR spectroscopy OR MRS OR PET OR "positron emission tomography"  OR radiomic* OR radiogenomic* OR "artificial intelligence" OR "machine learning"  OR "functional MRI" OR fMRI OR tractography OR "diffusion tensor imaging" OR DTI  OR "intraoperative MRI" OR iMRI OR "laser interstitial thermal therapy" OR LITT  OR "convection-enhanced delivery" OR CED OR "focused ultrasound"  OR "blood-brain barrier disruption" OR "blood brain barrier opening"  OR "intra-arterial" OR emboli?ation OR "stereotactic biopsy"  OR "image-guided" OR neurointervention*  ) ) AND PUBYEAR < 2027 | Language limited to English. Article, review, and conference-paper records were considered; conference abstracts were retained only for preliminary approaches lacking full publication. |
| **Google Scholar** | Initial search: December 2025 Final update: 31 July 2026 Coverage: all years through 31 July 2026 | Five topic-specific queries: 1. "glioblastoma" (MRI OR perfusion OR diffusion OR spectroscopy OR PET OR radiomics OR "artificial intelligence" OR "intraoperative MRI" OR LITT OR "convection-enhanced delivery" OR "focused ultrasound" OR "intra-arterial" OR embolization) 2. ("low-grade glioma" OR "IDH-mutant glioma") (MRI OR spectroscopy OR tractography OR PET OR radiomics OR LITT OR "molecular imaging") 3. ("brain metastases" OR "intracranial metastases") (MRI OR perfusion OR spectroscopy OR PET OR radiomics OR LITT OR embolization OR "image-guided") 4. ("primary central nervous system lymphoma" OR PCNSL) (MRI OR diffusion OR perfusion OR PET OR biopsy OR "intra-arterial" OR "blood-brain barrier disruption") 5. "meningioma" (MRI OR CT OR perfusion OR diffusion OR "somatostatin receptor PET" OR embolization OR LITT OR "artificial intelligence") | Searches were sorted by relevance. Duplicate records and clearly non-neuro-oncologic results were excluded. |
| **ClinicalTrials.gov** | Verified through 31 July 2026 | Targeted ClinicalTrials.gov searches: 1. Condition/disease: glioblastoma; Other terms: intra-arterial OR cetuximab OR blood-brain barrier disruption 2. Condition/disease: high-grade glioma; Other terms: temsirolimus OR intra-arterial 3. Condition/disease: primary central nervous system lymphoma; Other terms: intra-arterial OR osmotic blood-brain barrier disruption Trial records identified through included articles were verified directly by NCT number. | Used only to describe ongoing or preliminary interventions. Registrations were not treated as evidence of clinical efficacy. |
| **Professional-society guideline sources** | Verified through 31 July 2026 | Targeted searches of official professional-society sources: ACR, NCCN, ASCO, SNO, ASTRO, EANO, EHA, ESMO, EANM, RANO, and SNMMI. Search concepts combined each tumor type with relevant terms such as imaging, PET, response assessment, LITT, biopsy, embolization, intra-arterial therapy, and blood-brain barrier disruption. | The most current relevant guideline or consensus document available by the final search date was prioritized. Society webpages were used to identify the full guideline publication whenever available. |
| **Citation chaining and targeted updates** | Performed iteratively through 31 July 2026 | Reference lists of included guidelines, reviews, and key clinical studies were screened for additional eligible sources. Forward citation searching was used selectively for recent or foundational articles when needed to identify updates or related clinical evidence. | Recent guidelines and clinical studies were prioritized; older foundational studies were retained when directly relevant to classification, imaging interpretation, or procedural development. |

**Part B. Eligibility and study-selection framework**

| **Domain** | **Inclusion/approach** | **Exclusion/qualification** |
| --- | --- | --- |
| **Review scope** | Glioblastoma, low-grade glioma, brain metastases, primary central nervous system lymphoma, and meningioma. | Studies without a clear application to one of the five tumor groups or to neuro-oncologic care. |
| **Clinical focus** | Diagnosis, differential diagnosis, tumor characterization, treatment planning, image-guided intervention, response assessment, surveillance, patient selection, safety, and implementation. | Studies unrelated to diagnostic imaging or image-guided neuro-oncologic management. |
| **Publication types** | Peer-reviewed clinical studies, systematic reviews, narrative reviews, consensus statements, professional-society guidelines, and selected technical or translational studies relevant to emerging procedures. | Editorials, opinion pieces, commercial webpages, or non-peer-reviewed sources without substantive clinical or technical information. |
| **Preliminary or ongoing evidence** | Conference abstracts and trial registrations only when needed to describe preliminary or ongoing approaches for which full published results were unavailable; explicitly identified as preliminary. | Abstracts or registry records presented as evidence of clinical efficacy or comparative effectiveness. |
| **Population and setting** | Adult studies were emphasized. Pediatric studies were included selectively when they contributed to a limited evidence base and their restricted generalizability was stated. | Pediatric evidence generalized directly to adult disease without qualification. |
| **Language** | English-language publications. | Non-English publications. |
| **Date range** | Database inception through the final search date. | Publications after the final search date. |
| **Evidence prioritization** | Recent guidelines, consensus statements, systematic reviews, and clinical studies were prioritized; older foundational studies were retained when necessary. | Redundant or superseded sources when a more current primary or guideline source was available. |
| **Selection process** | Titles, abstracts, and full texts were assessed for relevance to the narrative aims. Reference lists of selected sources were reviewed for additional studies. | Formal dual-reviewer screening, protocol registration, and standardized risk-of-bias assessment were not performed because this was a narrative review. |
| **Data synthesis** | Evidence was synthesized qualitatively by tumor type, imaging modality, procedural indication, performing specialty, evidence status, limitations, and guideline position. | No pooled meta-analysis or formal comparative-effectiveness grading was performed. |
